# *Plasmodium falciparum* UvrD Helicase Translocates in 3′ to 5′ Direction, Colocalizes with MLH and Modulates Its Activity through Physical Interaction

**DOI:** 10.1371/journal.pone.0049385

**Published:** 2012-11-21

**Authors:** Moaz Ahmad, Abulaish Ansari, Mohammed Tarique, Akash Tripathi Satsangi, Renu Tuteja

**Affiliations:** Malaria Group, International Centre for Genetic Engineering and Biotechnology, Aruna Asaf Ali Marg, New Delhi, India; Institute of Enzymology of the Hungarian Academy of Science, Hungary

## Abstract

Malaria is a global disease and a major health problem. The control of malaria is a daunting task due to the increasing drug resistance. Therefore, there is an urgent need to identify and characterize novel parasite specific drug targets. In the present study we report the biochemical characterization of parasite specific UvrD helicase from *Plasmodium falciparum*. The N-terminal fragment (PfUDN) containing UvrD helicase domain, which consists of helicase motifs Q, Ia–Id, II, III and most of motif IV, and the C-terminal fragment (PfUDC1) containing UvrD helicase C terminal domain, consisting of remaining part of motif IV and motifs IVa–IVc and 161 amino acids of intervening sequence between motif IV and V, possess ssDNA-dependent ATPase and DNA helicase activities in vitro. Using immunodepletion assays we show that the ATPase and helicase activities are attributable to PfUDN and PfUDC1 proteins. The helicase activity can utilize the hydrolysis of all the nucleotide and deoxynucleotide triphosphates and the direction of unwinding is 3′ to 5′. The endogenous *P. falciparum* UvrD contains the characteristic DNA helicase activity. PfUDN interacts with PfMLH (*P. falciparum* MutL homologue) and modulates the endonuclease activity of PfMLH and PfMLH positively regulates the unwinding activity of PfUDN. We show that PfUvrD is expressed in the nucleus distinctly in the schizont stages of the intraerythrocytic development of the parasite and it colocalizes with PfMLH. These studies will make an important contribution in understanding the nucleic acid transaction in the malaria parasite.

## Introduction

Malaria is the major parasitic infection in many tropical and subtropical regions including India and thus remains a public health problem of enormous magnitude particularly in developing world. About 300–500 million people come in contact with malaria parasite every year and deaths from malaria parasite have been estimated to about 1–2 million each year [1]–[4]. *Plasmodium* spp. is obligate intracellular parasites, switching between an arthropod vector and their respective host where they undergo cycles of asexual reproduction in erythrocytes. During the last few years the situation has worsened in many ways, mainly due to malarial parasites becoming increasingly resistant to several anti-malarial drugs. Thus there is an urgent need to find alternate ways to control malaria and therefore it is necessary to identify new classes of anti-malarial drugs. Malaria pathogenesis is associated with the intracellular erythrocytic stage of the life cycle of the malaria parasite *Plasmodium falciparum* involving repeated rounds of invasion, growth, and schizogony.

Helicases are ubiquitous enzymes that catalyze the unwinding of energetically stable duplex DNA (DNA helicases) or duplex RNA secondary structures (RNA helicases). They play essential roles in basic cellular processes, such as DNA replication, repair, recombination, transcription and translation. One mechanism central to genomic stability and the control of mutagenesis is DNA repair, which removes potentially deleterious lesions through either damage reversal or damage excision. Helicases have roles in all the nucleic acid repair pathways such as nucleotide excision repair (NER), mismatch repair (MMR), base excision repair (BER), double strand break repair (DSBR) and also cross-link repair [5], [6]. DNA replication errors (base substitution mismatches and insertion-deletion loops) are primarily corrected by DNA MMR [7], [8]. Generally MMR, which is conserved from bacteria to eukaryotes involves the following steps: mismatch recognition, DNA nicking around the mismatch, mismatch strand removal and DNA synthesis to rectify the mistake. To maintain genomic stability in all organisms an active MMR system has to work efficiently to ensure the fidelity of chromosomal replication [9]. This is evident by the defects found in MMR genes in human cells which result in genomic instability and hereditary colon cancer [10]–[16].

**Figure 1:**
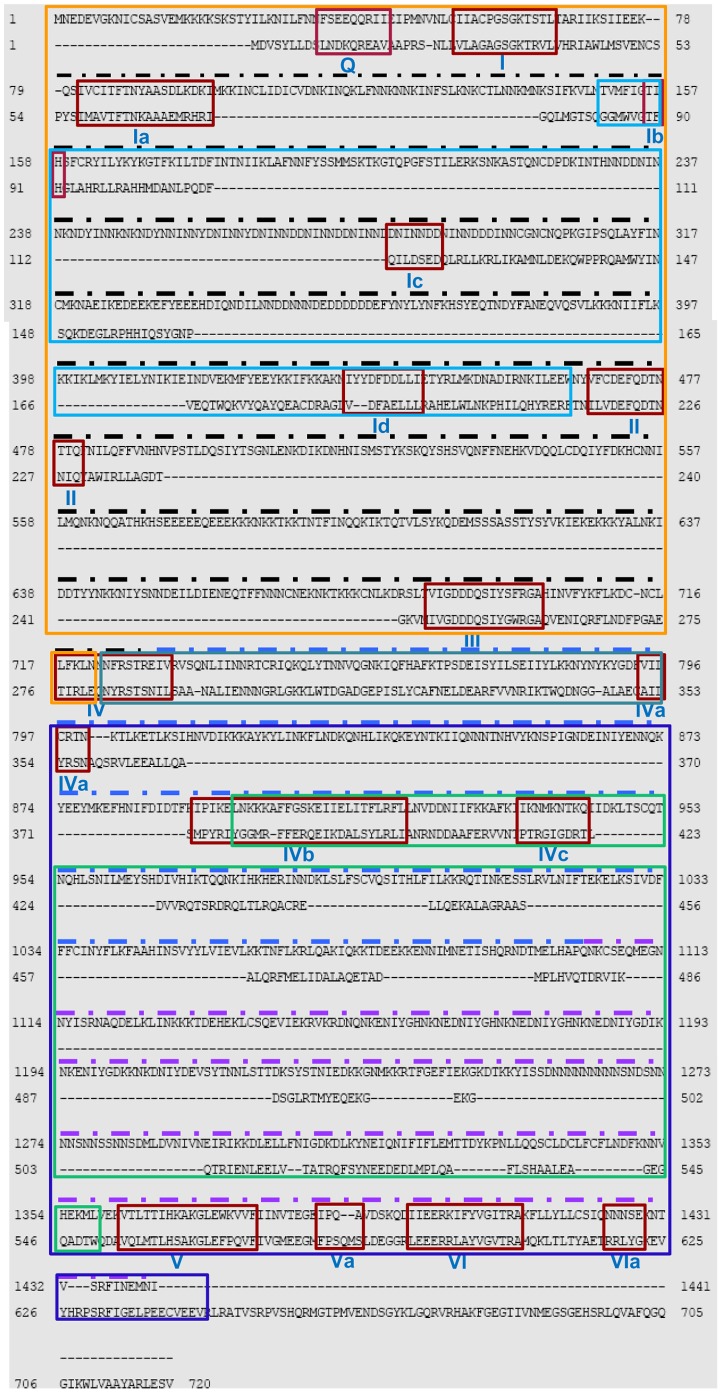
Comparison of amino acid sequence of *Plasmodium falciparum* (Pf) UvrD (1–1441) and *Escherichia coli* (*E. coli*) UvrD (1–720). The alignment was done using BLAST program (http://blast.ncbi.nlm.nih.gov/Blast). The conserved motifs are boxed in red color and the name of each motif (from Q to VIa) is written in roman numerals. The PlasmoDB number for *P. falciparum* UvrD sequence is PFE0705c and the accession number for *E. coli* UvrD sequence is BAA00048.1. The black, blue and purple dotted lines indicate the PfUDN, PfUDC1 and PfUDC2 fragments respectively. The orange box indicates the domain 1A (from amino acid 1–722) and the blue box inside it indicates the domain 1B (from amino acid 150–464). The purple box denotes the domain 2A (from amino acid 723–1441) and the green box inside it indicates the domain 2B (from amino acid 896–1359).

Malaria can be easily cured but the appearance of drug-resistance in *P. falciparum* is a major hindrance to the control of the disease [17], [18]. Although the mechanisms by which malaria parasites develop resistance to drugs are unclear, in other organisms, defects in DNA MMR have been linked to increased mutation rates and drug resistance. It is well established that the underlying cause of drug resistance in malaria is the development of specific genetic mutations. There are several sequences identified in PlasmoDB, that are homologous to genes involved in repair pathways from other organisms, indicating that this pathway is likely present in the parasite [19]. The most well characterized MMR pathway is of *Escherichia coli*. In *E. coli* UvrD is known to play an essential role in both the forms of DNA repair such as MMR [20] and the NER [21].

UvrD or DNA helicase II is a superfamily 1A helicase universally distributed across bacteria and extensively characterized [22]. It has also been reported that UvrD and its homologues such as PcrA and Rep represent one family known as PUR family and are targets for drug discovery because the deletion of PcrA is lethal in Staphylococcal species and *Bacillus subtilis* [23]. It has been shown that Mycobacterial UvrD2 is a DNA-dependent ATPase with 3′ to 5′ helicase activity [24]. In a recent work the UvrD from *Haemophilus influenza* (HiUvrD) and *Helicobacter pylori* (HpUvrD) have been shown to exhibit strong single-stranded DNA-specific ATPase and 3′–5′ helicase activities [25]. It is well known that the three helicases *Bacillus stearothermophilus* PcrA, *E. coli* Rep and *E. coli* UvrD are structurally similar and contain a two domain (1 and 2) structure with each domain made of two sub-domains (1A, 1B, 2A and 2B) and a C-terminal extension [26]–[28]. It has been shown that a truncated form of *E. coli* UvrD that lacks the C-terminal extension retains helicase activity on a variety of substrates [29].

The repair of misincorporated bases and damaged DNA is very important for maintenance of genomic integrity. It has been proposed recently that *P. falciparum* drug resistant parasites have defective MMR and this is the underlying mechanism in the development of antimalarial drug resistance [30]. Very little is known about DNA repair mechanisms in *P. falciparum* but due to the availability of its genome sequence direct comparison of potential DNA repair genes to their *E. coli* counterpart can be done. Previously we have reported that the parasite *P. falciparum* genome contains the homologues of major components of MMR complex such as UvrD helicase and MutL homologue (MLH) [22]. In a recent study we have reported the isolation and characterization of MLH from *P. falciparum* [31].

In the present study we describe the expression, purification and biochemical characterization of another main component of MMR complex, UvrD from *P. falciparum* 3D7 in detail. We found that the N-terminal (PfUDN, consisting of domain 1A and 1B) and the C-terminal (PfUDC1, consisting of first half of domain 2A and 2B) fragments of PfUvrD contain ssDNA-dependent ATPase and helicase activities. The *Km* values for helicase activity are 1.2±0.1 nM for PfUDN and 3.2±0.4 nM for PfUDC1 respectively. PfUDN is capable of unwinding the blunt end duplex DNA substrate also. The helicase activity is almost equal in all the NTPs and dNTPs tested and the polarity of unwinding is 3′ to 5′. The endogenous *P. falciparum* UvrD of ∼170 kDa contains the characteristic DNA helicase activity. We further show that PfUDN and PfMLH interact and positively regulate each other’s activity. Using immunofluorescence assays we report that both PfUvrD and PfMLH co-localize in the parasite *P. falciparum* 3D7 strain and are expressed in the schizont stages of intraerythrocytic development. These studies will advance our knowledge in the field of nucleic acid metabolism in the parasite.

**Figure 2:**
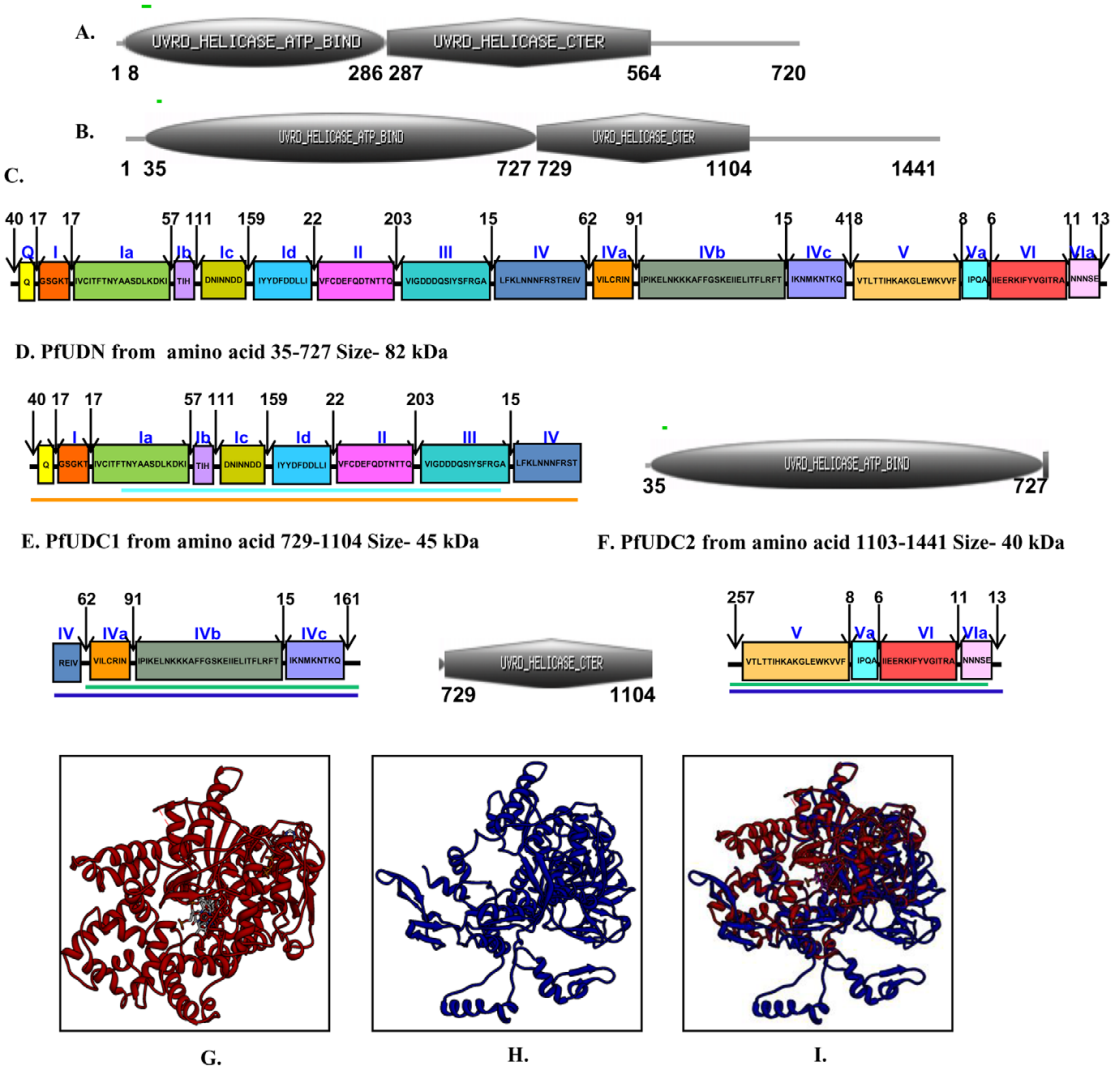
Schematic diagrams showing the domain organization. A, *E. coli* and **B,** *P. falciparum* UvrD helicases. Domain analysis was done using Scan Prosite at (http://expasy.org). The domain structure was taken from the results and used in the figures. UvrD helicase ATP binding and UvrD C-terminal domains are shown. The numbers show the amino acids spanning these motifs. **C,** The detailed domain organization of *P. falciparum* UvrD helicase. The conserved sequences of each domain are written inside the boxes. The text in blue refers to the names of various conserved domains and the numbers refer to the amino acids separating the various domains and the length of N- and C-terminal extensions. This figure is not drawn to scale. **D–F,** The detailed domain structure of PfUDN, PfUDC1 and PfUDC2 fragments of *P. falciparum* UvrD. The details are as in C. The colored lines are same as in Figure 1 and correspond to domain 1A, 1B, 2A and 2B present in PfUDN, PfUDC1 and PfUDC2 respectively. (**G–I**) Structure modeling. The PfUvrD full-length sequence was submitted to Swissmodel server and the structure was obtained. The molecular graphic images were produced using the UCSF Chimera package from the resource for Biocomputing, Visualization, and Informatics (http://www.cgl.ucsf.edu/chimera) at the University of California, San Francisco (supported by NIH P41 RR-01081). G. Template; H. full-length PfUvrD; I. superimposed image.

**Figure 3:**
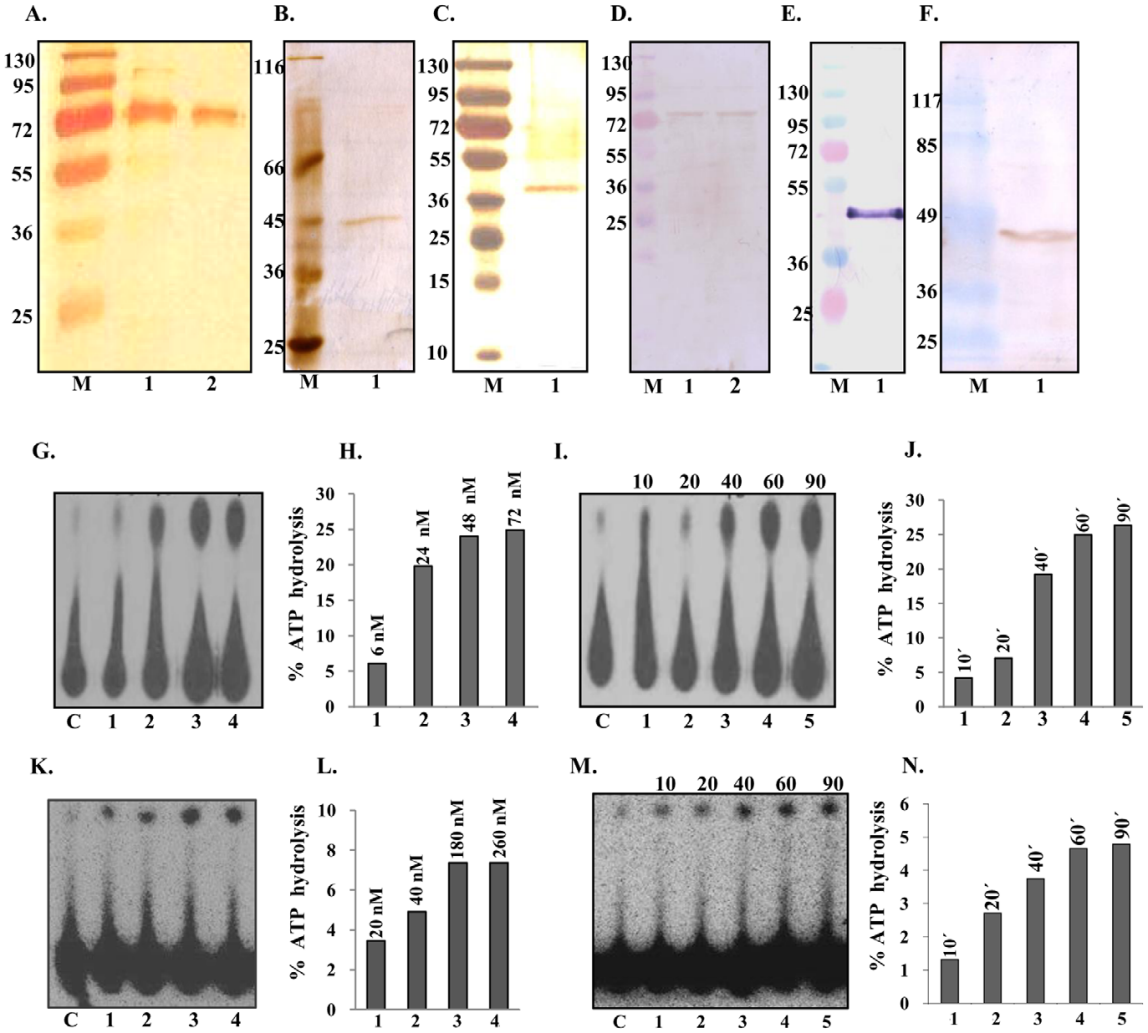
Purification and ATPase activity analysis. **A,** Silver-stained gel of purified PfUDN. Lane M contains the protein molecular weight marker and lane 1 and 2 contain 0.3 and 0.2 µg of the purified PfUDN. **B,** Silver-stained gel of purified PfUDC1. Lane M contains the protein molecular weight marker and lane 1 contains 0.2 µg of the purified PfUDC1. **C,** Silver-stained gel of purified PfUDC2. Lane M contains the protein molecular weight marker and lane 1 contains 0.2 µg of the purified PfUDC2. **D,** Western blot of purified PfUDN. Lane M contains the protein molecular weight marker and lane 1 and 2 contain 0.3 and 0.2 µg of the purified PfUDN. **E,** Western blot of purified PfUDC1. Lane M contains the protein molecular weight marker and lane 1 contains 0.2 µg of the purified PfUDC1. **F,** Western blot of purified PfUDC2. Lane M contains the protein molecular weight marker and lane 1 contains 0.2 µg of the purified PfUDC2. **G,** ATPase activity of purified PfUDN. Lane C, reaction without enzyme, Lanes 1–4, reactions with enzyme in the presence of ssDNA and Mg^2+^. **H,** The quantitative data of the autoradiogram in G. **I,** Time dependence of ATPase activity of PfUDN. The time of incubation in minutes is mentioned at the top of the autoradiogram and C is the control reaction without enzyme. **J,** The quantitative data of the autoradiogram in I. **K,** ATPase activity of purified PfUDC1. Lane C, reaction without enzyme, Lanes 1–4, reactions with enzyme in the presence of ssDNA and Mg^2+^. **L,** The quantitative data of the autoradiogram in K. **M,** Time dependence of ATPase activity of PfUDC1. The time of incubation in minutes is mentioned at the top of the autoradiogram and C is the control reaction without enzyme. **N,** The quantitative data of the autoradiogram in M.

## Materials and Methods

### Ethics Statement

The animal studies described in this study were approved by the ICGEB Institutional Animal Ethics Committee (IAEC Reference No. MAL-55). ICGEB is licensed to conduct animal studies for research purposes under the registration number 18/1999/CPCSEA (dated 10/1/99).

### Identification and Cloning of *P. falciparum* UvrD Gene

In order to clone the UvrD helicase from *P. falciparum*, the sequence was downloaded and analyzed in detail. The nucleotide sequence of PfUvrD is 4326 bases and it codes for a protein of 1441 amino acids. PCR amplification was done using genomic DNA as the gene is not interrupted by introns. Accordingly the primers PfUF1 (BamHI site) and PfUR1 (Xho I site), PfUF2 (BamHI site) and PfUR2 (Xho I site) and PfUF3 (BamHI site) and PfUR3 (Xho I site) were synthesized to clone the N-terminal and the C-terminal fragments C1 and C2. The *P. falciparum* UvrD helicase gene was amplified in three fragments using the following forward and the reverse primers.

1. PfUF1: 5′- GGGATCCAACTTTTCTGAGGAAC- 3′
2. PfUR1: 5′-GCTCGAGTGTACTTCGAAAATTATT- 3′
3. PfUF2: 5′-GGGATCCGAAATTGTTAGAGTTTC- 3′
4. PfUR2: 5′-GCTCGAGATTTTGAGGTGCATGT- 3′
5. PfUF3: 5′- GGGATCCCAAAATAAATGTAGCG 3′
6. PfUR3: 5′- GCTCGAGTATATTCATTTCATTAAT 3′

The N-terminal fragment is 2079 bases and codes for PfUDN fragment from amino acid 35–727, the C1 fragment is 1128 bases and codes for PfUDC1 fragment from amino acid 729–1104 and the C2 fragment is 1020 bases and codes for PfUDC2 fragment from amino acid 1103–1441 respectively. The PCR conditions used were 95°C for 1 minute, 54°C for 1 min and 72°C for 2 min for the amplification of N terminal fragment. For the amplification of the C terminal fragments the extension was done only for 1.5 minutes. This was repeated for a total of 35 cycles and at the end one elongation was done at 72°C for 12 min. The PCR products were gel purified using Qiagen gel extraction kit and cloned into the pGEM-T easy vector from Promega using T-A cloning (Madison, WI, USA) and the clones were sequenced by dideoxy sequencing reactions (Macrogen, Korea). The nucleotide sequence was submitted to GenBank and the accession number for the PfUDN fragment is FJ588849, for the PfUDC1 fragment is FJ455133 and for the PfUDC2 fragment is JN016528.

Using BamHI and XhoI enzymes (New England Biolabs, Beverly, MA, USA) all the fragments were excised from pGEMT easy clones, gel purified and subsequently cloned into pET28a+ expression vector (Novagen, Madison, WI, USA) at the appropriate sites. For protein expression, the clones were transformed into BL21 (DE3) pLysS cells. 1% of the overnight grown primary culture was inoculated in 500 ml LB (Luria Broth) and allowed to grow at 37°C. At OD 0.6 the culture was induced with 1 mM IPTG and then again allowed to grow for another 4–6 hours. The harvested cells were lysed by using lysis buffer of pH 7.8 (20 mM Tris-HCl, 250 mM NaCl, 0.1% Tween 20, 0.1% Triton 100 and the protease inhibitor cocktail from Sigma, St. Louis, MO, USA) and subsequently the cells were sonicated to lyse maximum number of cells. After centrifugation the soluble fraction was allowed to bind to pre-equilibrated Ni-NTA (Qiagen, GmbH, Germany) resin for one hour at 4°C. The column was first washed with the wash buffer (lysis buffer without detergent with 25 mM imidazole). The bound His-tagged proteins were eluted with varying (100–150 mM) concentration of imidazole in the protein buffer (20 mM Tris–HCl pH 8.0, 250 mM NaCl, 10% (v/v) glycerol and protease inhibitor cocktail from Sigma, St. Louis, MO, USA) and was checked for purity by SDS-PAGE (10% (w/v) polyacrylamide gel) and silver staining using slight modifications of the standard protocol [32]. The slight modification included extensive washing of the gel after fixation and this step reduces the background and increases the sensitivity of the stain.

**Figure 4:**
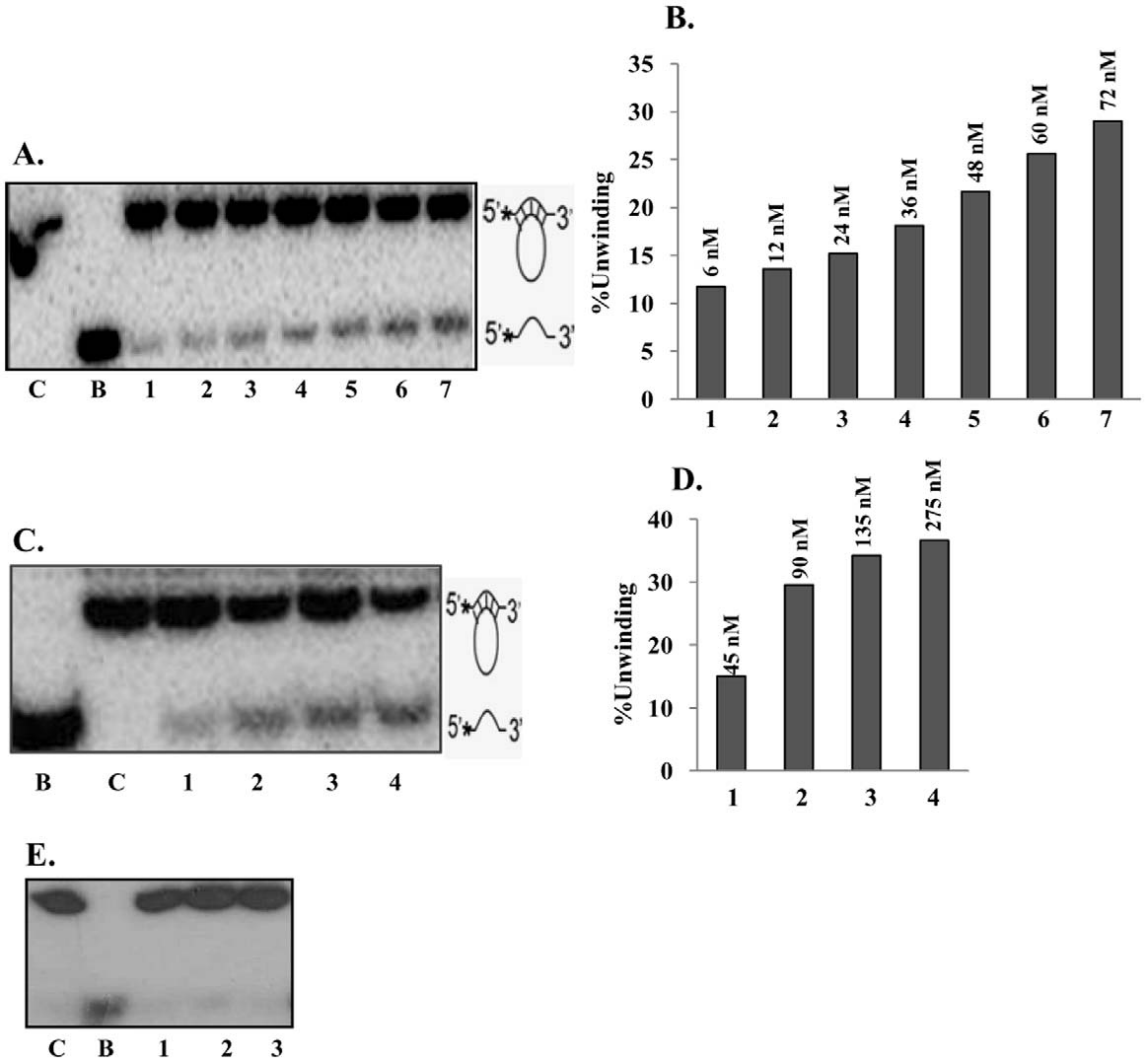
Helicase activity analysis. **A,** Helicase activity of purified PfUDN, lanes 1–7 contain increasing concentration of PfUDN, C is reaction without enzyme and B is heat denatured substrate. **B,** The quantitative enzyme activity data from the autoradiogram in A are shown and the concentration of PfUDN is also written. **C,** Helicase activity of purified PfUDC1, lanes 1–4 contain increasing concentration of PfUDC1, C is reaction without enzyme and B is heat denatured substrate. **D,** The quantitative enzyme activity data from the autoradiogram in C are shown and the concentration of PfUDC1 is also written. **E,** Helicase activity of purified PfUDC2, lanes 1–3 contain increasing concentration of PfUDC2, C is reaction without enzyme and B is heat denatured substrate.

**Figure 5:**
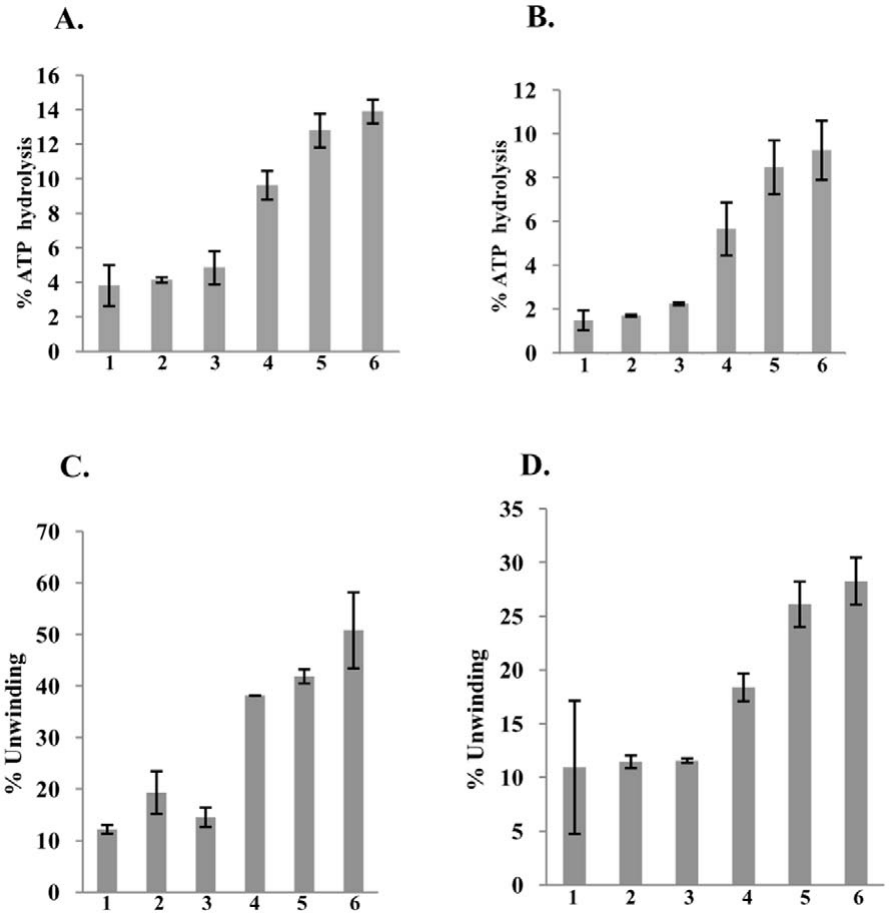
Immunodepletion of ATPase and helicase activities. **A and B,** Quantitative data of immunodepletion of ATPase activity. **A,** Lanes 1–3, reactions with increasing concentration of PfUDN pretreated with immune IgG and lanes 4–6, reactions with increasing concentration of PfUDN pretreated with pre-immune IgG. **B,** Lanes 1–3, reactions with increasing concentration of PfUDC1 pretreated with anti-his IgG and lanes 4–6, reactions with increasing concentration of PfUDC1 pretreated with pre-immune IgG. Each bar represents the mean percentage ± SD of two different experiments. **C and D,** Quantitative data of immunodepletion of helicase activity. **C,** Lanes 1–3, reactions with increasing concentration of PfUDN pretreated with immune IgG and lanes 4–6, reactions with increasing concentration of PfUDN pretreated with pre-immune IgG. **D,** Lanes 1–3, reactions with increasing concentration of PfUDC1 pretreated with anti-his IgG and lanes 4–6, reactions with increasing concentration of PfUDC1 pretreated with pre-immune IgG. Each bar represents the mean percentage ± SD of two different experiments.

### Western Blot Analysis

For western blotting, the proteins were separated by SDS-PAGE and transferred electrophoretically to nitrocellulose membrane as described [32]. After blocking with 3% skimmed milk in TBST (Tris buffered saline with 0.05% Tween 20), the membrane was incubated with the appropriate primary antibody (Penta-His from Qiagen, GmbH, Germany) for 3 h at room temperature. After washing, the blot was incubated with the appropriate secondary antibody coupled to alkaline phosphatase (Sigma, St. Louis, MO, USA) and developed using 5-Bromo-4-Chloro-3-Indolyl Phosphate and Nitro Blue Tetrazolium obtained from Sigma.

### Generation of Polyclonal Antisera

Purified PfUDN was used for the preparation of antibodies in mice using the standard protocols [32]. The polyclonal antibodies were purified as IgG fractions using protein A-Sepharose as described [32].

### ATPase Assay

The ATPase reaction was performed in the buffer (20 mM Tris-HCl, pH 8.0, 8 mM DTT, 1.0 mM MgCl_2_, 20 mM KCl and 16 µg/ml BSA) for 1 hour at 37°C in the presence of purified PfUDN, PfUDC1 or PfUDC2 and 10 ng of M13 mp19 ssDNA and a mixture of [γ-^32^P] ATP (∼17 nM) and 1 mM cold ATP. The products were separated by thin layer chromatography (TLC) [33]–[35] and the plate was exposed to hyper film for autoradiography or scanned on phosphoimager. The quantitation was done using IMAGE j/geldoc software (http://rsbweb.nih.gov/ij/). For the concentration curve analysis different concentrations of PfUDN (from 6 to 72 nM) and PfUDC1 (from 20 to 260 nM) proteins were used. The time course analysis was performed with a fixed concentration of PfUDN or PfUDC1 and time duration ranging from 10 to 90 minutes. The quantitation was done using IMAGE j/geldoc software (http://rsbweb.nih.gov/ij/) and percentage of ATP hydrolysis was plotted as the bar diagram.

**Figure 6:**
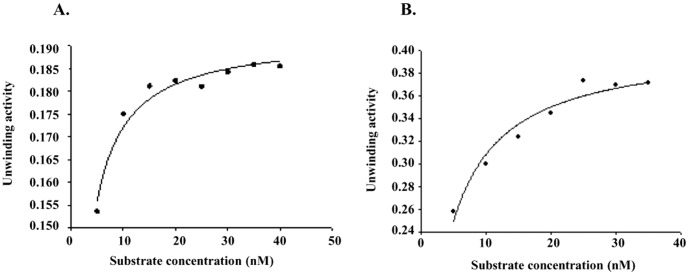
Kinetics of helicase activity. Km and Vmax of helicase activity of **A,** PfUDN and **B,** PfUDC1. The helicase reactions were carried out as reported in materials and methods section and K_m_ and V_max_ were calculated from the plot.

### Preparation of DNA Helicase Substrate and Helicase Assay

The helicase activity of PfUDN, PfUDC1 and PfUDC2 was determined by the standard strand displacement assay using the partially duplex substrate and the method described previously [33]–[35]. The normal substrate (substrate 1, Table S1) used in this study consisted of a ^32^P-labelled 47-mer DNA oligodeoxynucleotide annealed to M13mp19 phage ssDNA to create a partial duplex. At both the 5′ and 3′ ends, this oligodeoxynucleotide contains 15 base-pairs of non-complementary region. 10 ng of the oligodeoxynucleotide was labelled at 5′-end with T4 polynucleotide kinase (PNK) (5 U) (New England Biolabs) and 1.85 MBq of [γ-^32^P]ATP (specific activity 222 TBq/mmol) using the standard PNK buffer (New England Biolabs) at 37°C for one hour. The labeled oligodeoxynucleotide was then annealed with 0.5 µg of single-stranded circular M13mp19 (+) phage DNA using standard annealing buffer (20 mM Tris-HCl, pH 7.5, 10 mM MgCl_2_, 100 mM NaCl, 1 mM DTT) by heating at 95°C for 1 min, transferring immediately to 65°C for 2 min and then cooling slowly to room temperature. The non-hybridized oligodeoxynucleotide was removed using gel filtration through a Sepharose 4B column (Pharmacia, Sweden). The reaction mixture (10 µl) containing appropriate buffer (20 mM Tris-HCl, pH 8.0, 8 mM DTT, 1.0 mM MgCl_2_, 20 mM KCl and 16 µg/ml BSA), the ^32^P-labeled helicase substrate (1000 cpm/10 µl) (Table S1) and the purified protein fractions to be assayed was incubated at 37°C for 60 min. The substrate and products were separated by electrophoresis on a nondenaturing 12% or 15% (for the blunt end substrate) PAGE, dried, and the gel was exposed to hyper film for autoradiography or scanned on phosphoimager and both the substrate and unwound DNA bands were quantified. The quantitation was done using IMAGE j/geldoc software (http://rsbweb.nih.gov/ij/) and the percent unwinding was plotted as the bar diagram.

### Immunodepletion Assay

For this assay aliquots of the purified PfUDN and PfUDC1 were incubated with IgG purified from anti-preimmune and/or anti-PfUDN or anti-His antisera (for PfUDC1) respectively at 0°C for 60 min. The antigen–antibody complexes were removed by the addition of protein A Sepharose beads. The supernatants were used for the ATPase and helicase activity analysis using substrate 1 (Table S1) in the same way as described above. The quantitation was done using IMAGE j/geldoc software (http://rsbweb.nih.gov/ij/) and the percentage of ATP hydrolysis and percent unwinding was plotted as the bar diagram and each bar indicates the mean percentage ± SD (standard deviation).

**Figure 7:**
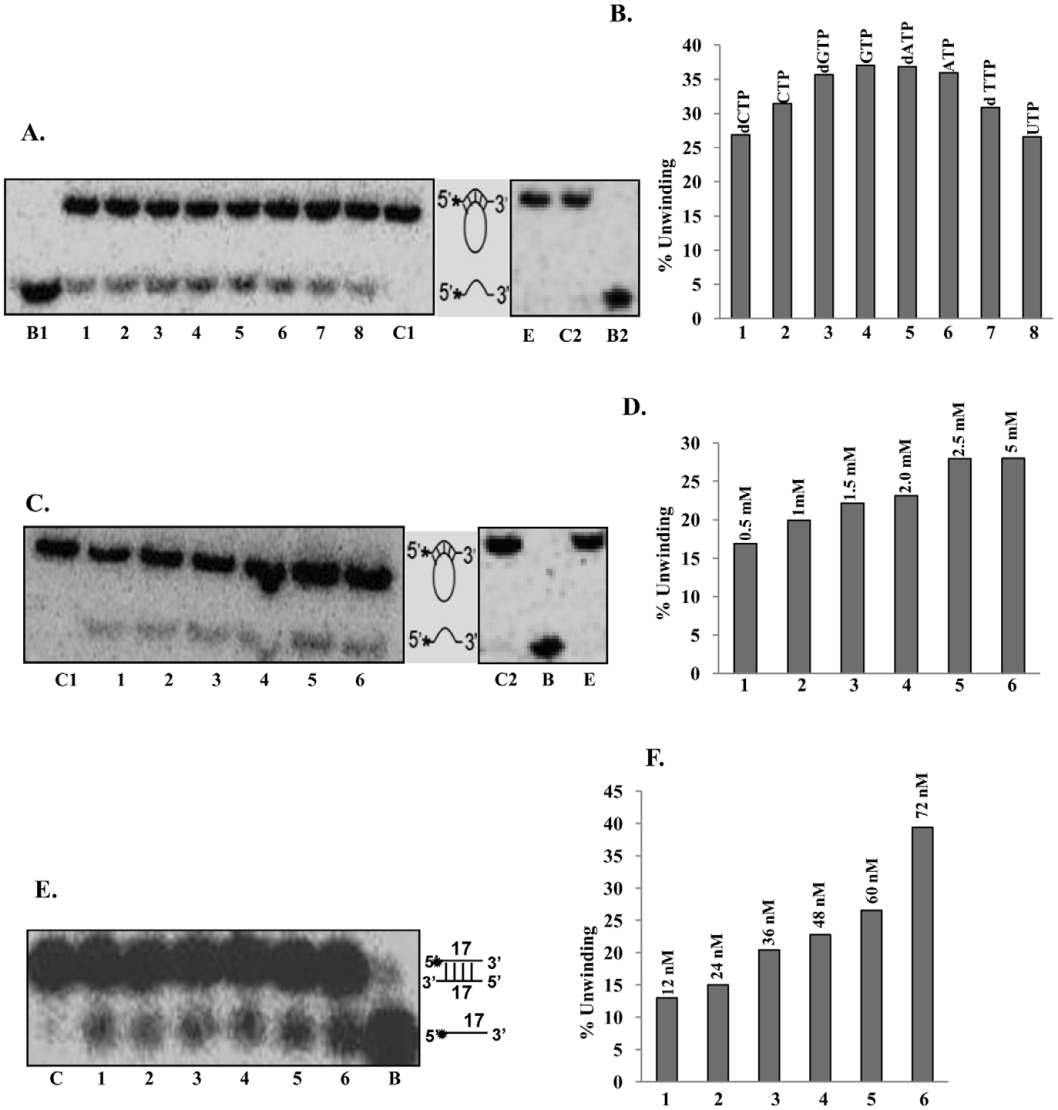
Further characterization of helicase activity. **A,** Nucleotide requirement of helicase activity of PfUDN. Helicase activity of PfUDN in the presence of lane 1, dCTP, lane 2, CTP, lane 3, dGTP, lane 4, GTP, lane 5, dATP, lane 6, ATP, lane 7, dTTP and lane 8, UTP. Lane E is enzyme reaction of PfUDN in the absence of any NTP or dNTP. C1 and C2 are reactions without enzyme and B1 and B2 are heat denatured substrates respectively. **B,** The quantitative enzyme activity data from the autoradiogram in A are shown and the various NTPs/dNTPs used are also written. **C,** Helicase activity of PfUDN using varying concentration of ATP. Lane 1, 0.5, lane 2, 1.0, lane 3, 1.5, lane 4, 2.0, lane 5, 2.5 and lane 6, 5.0 mM ATP. C1 and C2 are reactions without enzyme and B is heat denatured substrate respectively. Lane E is enzyme reaction in the absence of ATP. **D,** The quantitative enzyme activity data from the autoradiogram in C are shown and the concentration of ATP used is also written. **E,** Unwinding activity of PfUDN with blunt end duplex substrate. The helicase reaction was performed under standard assay conditions; the structure of the substrate used and the autoradiogram of the gel are shown. Asterisk (*) denotes the ^32^P-labeled end. Lanes 1–6 are the reactions with increasing concentration of enzyme, C is no enzyme control and B is heat-denatured substrate, respectively. **F,** The quantitative enzyme activity data from the autoradiogram in E are shown and the concentration of PfUDN used is also written.

### Determination of Km and Vmax

Helicase assay reactions for PfUDN and PfUDC1 were performed using the normal substrate (substrate 1, Table S1) of different concentrations (5–40 nM) in a standard reaction buffer (20 mM Tris-HCl, pH 8.0, 8 mM DTT, 1.0 mM MgCl_2_, 20 mM KCl and 16 µg/ml BSA). The amount of dsDNA and unwound ssDNA was quantified from the autoradiogram using ImageJ software (http://rsbweb.nih.gov/ij/) and used for the Km and Vmax calculations.

### Preparation of Blunt end DNA Helicase Substrate

The sequence of 17 mer oligodeoxynucleotide used for making the blunt end duplex substrate (substrate 2, Table S1) is as follows 5′-GTTTTCCCAGTCACGAC-3′. This was labeled at 5′ end using the method described above and was annealed to its complementary oligodeoxynucleotide with the sequence 5′-GTCGTGACTGGGAAAAC-3′. The substrate was purified and used for the assay using the method described above. The amount of dsDNA and unwound ssDNA was quantified from the autoradiogram using ImageJ software (http://rsbweb.nih.gov/ij/) and the percent unwinding was plotted as the bar diagram.

**Figure 8:**
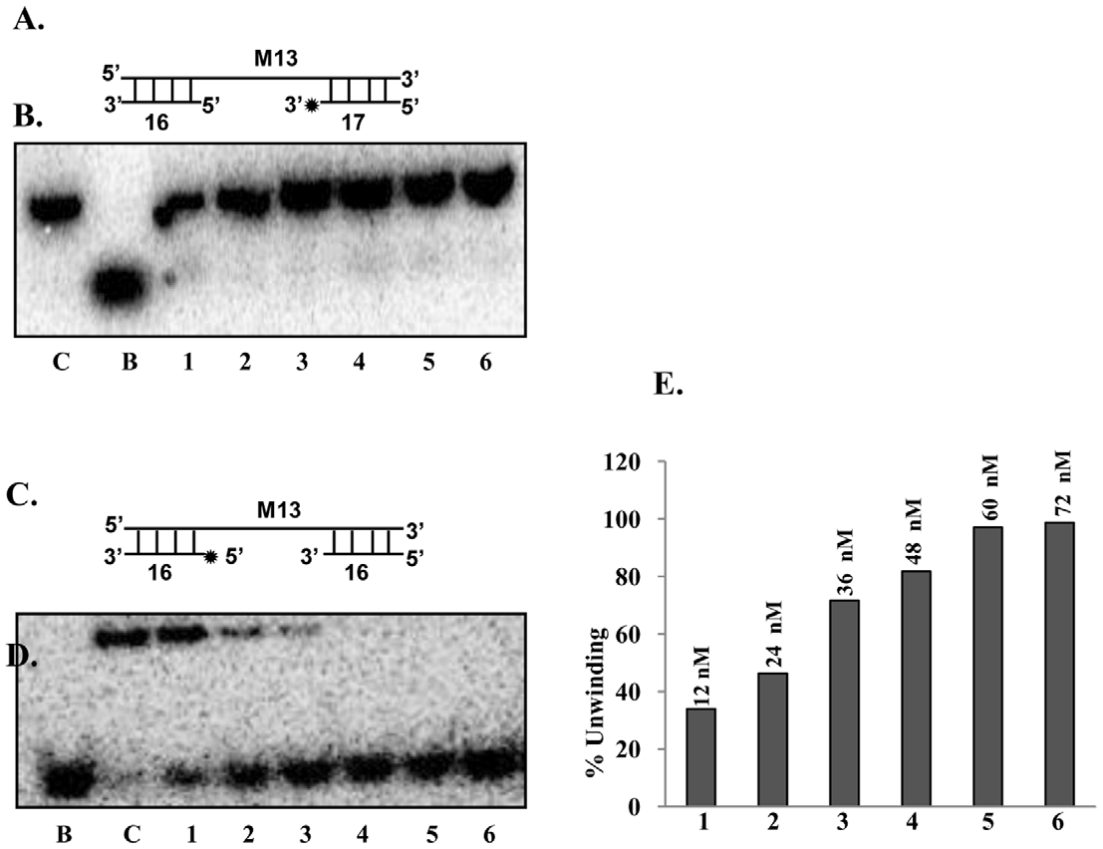
Direction of unwinding by PfUDN. **A,** The structures of the linear substrate for the 5′ to 3′ direction (substrate 3A, Tale S1). An asterisk (*) denotes the ^32^P-labeled end. **B,** Helicase activity using the substrate 3A (Tale S1) shown in A. Lane C is the reaction without enzyme and lane B is the heat-denatured substrate. Lanes 1–6 are reactions with increasing concentration of PfUDN. **C,** The structure of the linear substrate for the 3′ to 5′ direction (substrate 3B, Tale S1). An asterisk (*) denotes the ^32^P-labeled end. **D,** The helicase activity using the substrate 3B (Tale S1) shown in C. Lane C is the reaction without enzyme and lane B is the heat-denatured substrate. Lanes 1–6 are reactions with increasing concentration of PfUDN. **E,** The quantitative enzyme activity data from the autoradiogram in D are shown and the concentration of PfUDN used is also written.

### Preparation of Direction Specific Substrates

For constructing a 5′ to 3′ direction-specific substrate (substrate 3A, Table S1), the oligodeoxynucleotide 32-mer (5′-TTCGAGCTCGGTACCCGGGGATCCTCTAGAGT-3′) was first annealed to M13mp19 ssDNA using annealing buffer (20 mM Tris-HCl, pH 7.5, 10 mM MgCl_2_, 100 mM NaCl, 1 mM DTT) and then labeled at 3′-OH end in appropriate buffer with 50 µCurie [α-^32^P]dCTP and 5 units of DNA polymerase I (large fragment) at 23°C for 20 min. The incubation was continued for an additional 20 min at 23°C after increasing the dCTP to 50 mM using unlabelled dCTP. This resulting duplex substrate was digested with SmaI and purified by gel filtration through 1 ml Sepharose 4B. The substrate consisting of long linear M13mp19 ssDNA with short duplex ends for 3′ to 5′ unwinding (substrate 3B, Table S1) was prepared by first 5′-end labeling of 32-mer oligodeoxynucleotide and then annealing with M13mp19 ssDNA as described above. The annealed substrate was digested with SmaI and purified by gel filtration through 1 ml of Sepharose 4B.

### Purification of Endogenous UvrD Protein from Parasite Lysate

The endogenous UvrD protein was recovered by immunoaffinity purification from *P. falciparum* 3D7 strain. *P. falciparum* 3D7 strain was cultured with human erythrocytes (4% hematocrit) in RPMI media supplemented with 10% O+ human serum using standard protocol [36]. The basic protocol of the kit was slightly modified to minimize the nonspecific binding and elution of protein in the active state (as per recommendation of the Pierce kit protocol). *P. falciparum* 3D7 parasite pellet was suspended in the lysis buffer (Pierce, Thermo Scientific) containing 25 mM Tris-HCl pH 7.2, 150 mM NaCl, 1 mM EDTA, 1% NP-40 and 5% glycerol. After repeated freeze thaw, the lysate was centrifuged at ∼13000 × g for 10 minute to pellet the cell debris. Before performing the immunoprecipitation, soluble fraction of the parasite lysate was first precleared using control agarose resin (Pierce, Thermo Scientific) then with preimmune IgG-protein A agarose column to minimize the non specific protein binding. The purified IgGs from preimmune serum or anti-PfUDN mouse antiserum were diluted 1∶1 with 1x coupling buffer and were incubated in the column containing protein A/G+ agarose. After one hour of binding, the beads were washed with 1x coupling buffer containing 10 mM sodium phosphate pH 7.2 and 150 mM NaCl. The bound IgG-protein A/G+ agarose was cross linked in the coupling buffer containing 450 µM DSS (disuccinimidyl substrate) following the crosslinked immunoprecipitation kit protocol (Pierce, Thermo Scientific). The cross-linked beads were washed twice with elution buffer to remove non-crosslinked IgGs and quenching the cross linking reaction. Before incubating the cross linked beads with precleared parasite lysate, the beads were washed twice with lysis buffer as per instructions in Pierce® crosslink immunoprecipitation kit protocol. Equal amounts of the precleared parasite lysate was incubated overnight (4°C) with both preimmune and anti-PfUDN columns separately. The column was washed twice with lysis buffer then with 1X TBS (Tris buffered saline) to remove the detergent from column. The beads were subsequently washed with conditioning buffer (neutral pH buffer). The bound proteins were eluted in neutral pH buffer ((Pierce, Thermo Scientific) and quickly buffer was exchanged with Tris buffer (20 mM Tris-HCl, 150 mM NaCl with protease inhibitor cocktail) by using 10 kDa cutoff amicon filters (Pall Life Sciences). All the column elutes (preimmune column and anti-PfUDN column) were checked with SDS-PAGE coupled western blot analysis and used for further biochemical assays. Before enzymatic assay the elutes were passed through protein A agarose spin columns to trap the IgG and the flowthrough was used for the assay. For western blot analysis the proteins were transferred onto nitrocellulose membrane. After overnight blocking in 5% skimmed milk in TBST (Tris buffered saline with.05% Tween 20), the membrane was first incubated overnight at 4°C with a 1∶500 dilution of the purified IgG of anti-PfUDN and then incubated with the anti-mouse secondary antibodies (Sigma, St. Louis, MO, USA) coupled to horse radish peroxidase. The blot was developed using Sigma Fast™ DAB (3, 3-diaminobenzidine tetrahydrochloride) with urea enhancer tablets (St. Louis, MO, USA) according to the manufacturer’s instructions.

### Helicase Assays with the Endogenous PfUvrD

The helicase assays were performed with increasing volume of the elutes obtained from the pre-immune and anti-PfUDN columns described in the previous section. The normal substrate (substrate 1, Table S1) and the method described in the earlier section was used.

**Figure 9:**
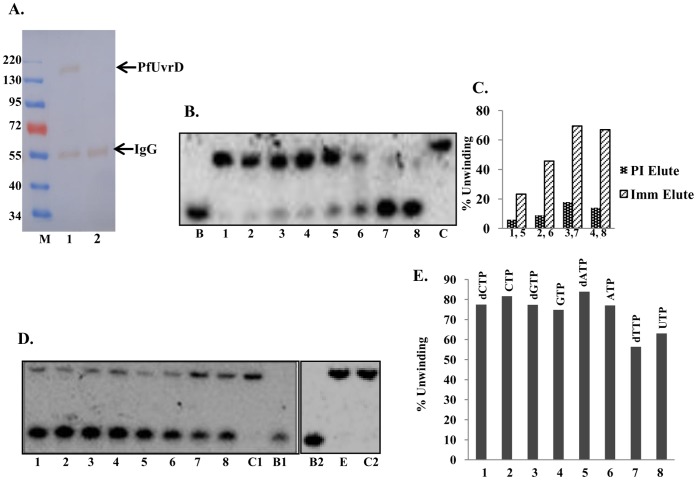
Immunoprecipitation and activity analysis of endogenous *P. falciparum* UvrD protein. **A.** Western blot. Lane M is prestained marker. Lane 1 is immunoprecipitate using anti-PfUDN IgG, and lane 2 is immunoprecipitated sample using preimmune IgG. The IgGs were crosslinked to minimize the elution but little amount of heavy chain was detected in the western blot. PfUvrD protein band is marked with arrow. **B.** Helicase activity. The helicase assay was done using the normal substrate (substrate 1, Tale S1). Lanes 1–4, reactions with increasing concentration of elute using preimmune IgG and lanes 5–8, reactions with increasing concentration of elute using anti-PfUDN IgG. Lane B is boiled control and lane C is control without protein. **C.** The quantitative enzyme activity data from the autoradiogram in B are shown. The numbers correspond to the lanes in B. **D.** Nucleotide requirement of helicase activity of endogenous PfUvrD. Helicase activity of endogenous PfUvrD in the presence of lane 1, dCTP, lane 2, CTP, lane 3, dGTP, lane 4, GTP, lane 5, dATP, lane 6, ATP, lane 7, dTTP and lane 8, UTP. C1 and C2 are reactions without enzyme and B1 and B2 are heat denatured substrates respectively. Lane E is enzyme reaction of endogenous PfUvrD in the absence of any NTP or dNTP. **E,** The quantitative enzyme activity data from the autoradiogram in D are shown and the various NTPs/dNTPs used are also written.

### Helicase Assay in the Presence of PfMLH

Helicase assay was performed using the previously characterized purified recombinant synthetic PfMLH [31], catalytically inactive N-terminal (PfMLHN), C-terminal (PfMLHC) [31] and the heat inactivated PfMLH and PfUDN. The strand displacement assay was used with both normal (substrate 1, Table S1) and direction-specific substrates (substrate 3B, Table S1). Both substrate and unwound DNA bands were quantified from the autoradiogram using ImageJ software (http://rsbweb.nih.gov/ij/) and the mean percentage of unwinding was plotted in the bar diagram and each bar indicates the mean percentage ± SD (standard deviation).

### Endonuclease Assay in the Presence of PfUDN and PfUDC1

The endonuclease assay reaction (10 µl) was performed as described earlier [31] by incubating 20 nM of PfMLH with 50 ng of pBR322 DNA in a buffer (buffer 4, New England Biolabs) containing 50 mM potassium acetate, 20 mM Tris–acetate, pH 7.9, 10 mM magnesium acetate and 1 mM dithiothreitol at 37°C for 1 hour. The reactions were stopped with loading dye (0.5% SDS, 50 mM EDTA, 40% glycerol, 0.1% bromophenol blue, 0.1% xylene cyanol) and the products were analyzed by 1% agarose gel electrophoresis and the bands were quantitated by ImageJ software (http://rsbweb.nih.gov/ij/). The percentage of nicked DNA was plotted in the bar diagram. Each bar indicates the mean percentage ± SEM (standard error mean). To investigate the effect of PfUDN and PfUDC1 on the endonuclease activity of PfMLH, the assay was performed with 20 nM PfMLH along with 15 nM PfUDN or PfUDC1. The negative control reactions without protein were also performed to compare the results.

### Enzyme-linked Immunosorbent Assay (ELISA) for Protein-protein Interaction Study

The 96 well ELISA plates were coated with fixed concentration (10 ng) of purified PfMLH diluted in bicarbonate/carbonate coating buffer (100 mM NaHCO_3_ and Na_2_CO_3_, pH 9.6), overnight at 4°C in 100 µl volume. The plate was emptied and washed three times with washing buffer phosphate buffered saline (PBS containing 0.05% Tween 20) and blocked for 2 hour with 5% non-fat milk in PBS. The blocking buffer was removed and the wells were washed 3 times with same washing buffer. Varying concentrations of the interacting proteins (from 2.5 ng to 30 ng) were added and the incubation was continued at 37°C for further 2 hour. The plate was emptied and washed three times with washing buffer (PBS containing 0.05% Tween 20). The assembly of the protein complex in the wells was then assessed through the use of polyclonal antibodies at 1∶15000 dilutions. The incubation with antibodies was done for 2 hour at 37°C. The plate was washed three times with washing buffer to remove the unbound antibodies. The plate was further incubated with 1∶3000 dilution of horse radish peroxidase (HRP) conjugated secondary antibody for 2 hours at 37°C. The unbound antibody was removed by washing the wells with washing buffer. The binding of the secondary antibody to the protein complex was then detected by applying the o-Phenylenediamine (OPD) substrate (Sigma, St. Louis, MO, USA) in appropriate buffer. The reaction was stopped and the plate was read at 490 nm using an ELISA reader and each bar indicates the mean percentage ± SD (standard deviation).

### In vitro Protein-protein Interaction Using Immunoaffinity Column

Protein A sepharose columns were prepared using anti-PfUDN and anti-preimmune IgGs. Purified anti-PfUDN IgGs and preimmune IgGs were allowed separately to bind onto protein A Sepharose column and both the columns were washed 3–4 times with buffer (20 mM Tris-HCl, pH 8.0, 135 mM NaCl and protease inhibitor cocktail) to remove the unbound antibody. For the assay 10 µg of each protein i.e. PfUDN and PfMLH in buffer (20 mM Tris-HCl, pH 8.0, 135 mM NaCl and protease inhibitor cocktail from Sigma, St. Louis, MO, USA) was mixed and allowed to interact for 1 hour at 4°C. Equal amount of this mixture was incubated with protein A Sepharose coupled to preimmune or anti-PfUDN IgG column for 1 hour at 4°C on shaker. After four washes with the buffer (20 mM Tris-HCl, pH 8.0, 135 mM NaCl and protease inhibitor cocktail from Sigma, St. Louis, MO, USA), the bound protein was eluted separately from both the columns with 100 mM glycine pH 3.0 and each fraction of both the elutes was run on SDS/PAGE and western blot analysis was performed. The anti-PfUDN IgG protein A Sepharose column elute was run in duplicate in order to probe with both the antibodies separately (anti-PfUDN and anti-PfMLH). The blots were further probed with appropriate secondary antibodies coupled with horse radish peroxidase (HRP) and were developed by Sigma fast® DAB tablets according to manufacturer’s instructions.

**Figure 10:**
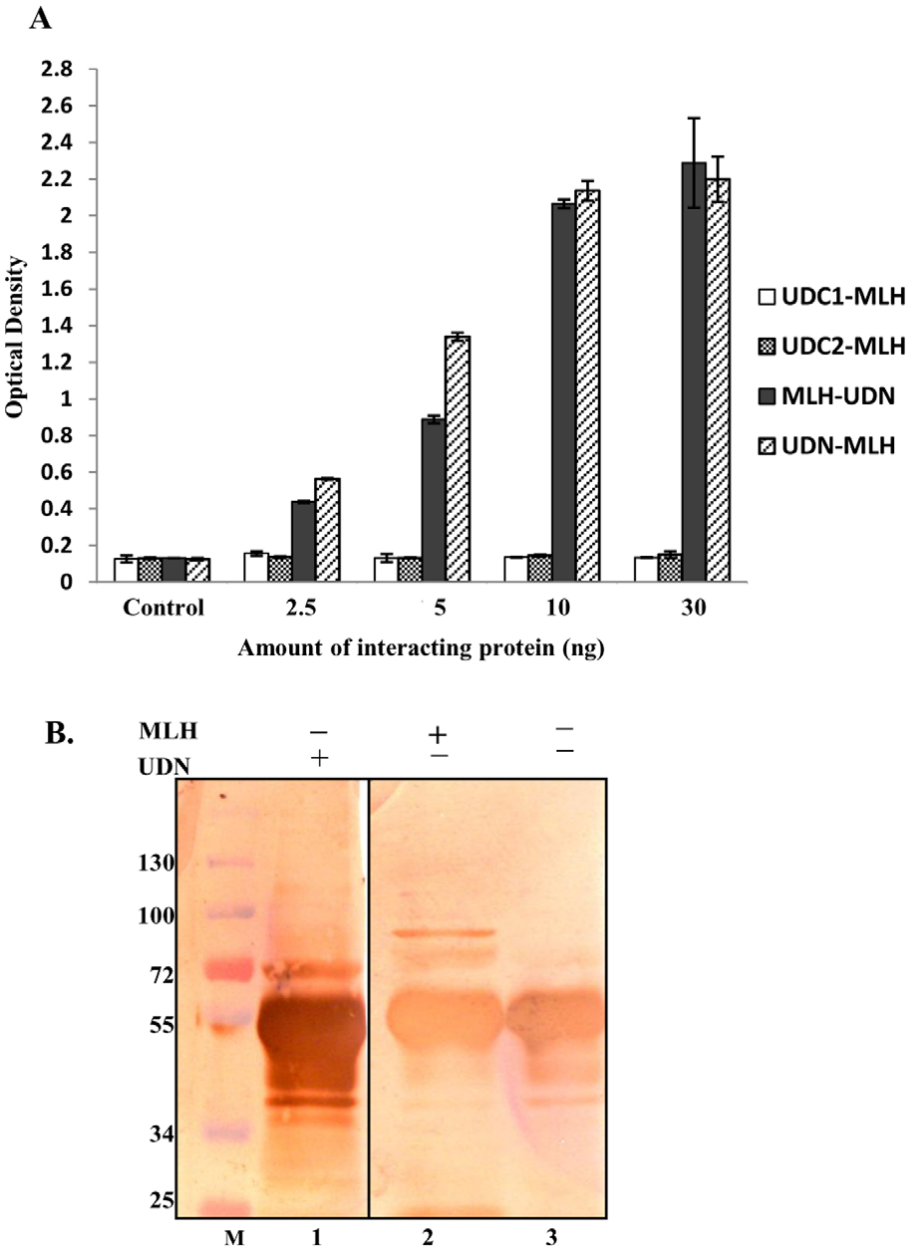
Protein-protein interaction study. A, ELISA based protein-protein interaction study of PfMLH with PfUDN, PfUDC1, and PfUDC2. The proteins coated such as PfUDC1, PfUDC2, PfMLH and PfUDN are written in the first column followed by the interacting proteins. The interaction was done as reported in materials and methods section. This experiment was repeated at least three times and the quantitative data are shown. **B,** Western blots of protein-protein interaction. Lane 1 and 2 are eluted fractions of in vitro immunoprecipitates of anti PfUDN IgG protein A sepharose column, lane 3 is immunoprecipitate of preimmune IgG protein A sepharose column. Lane 1 is probed with anti-PfUDN antibody; lanes 2 and 3 were probed with anti-MLH antibody.

### Immunofluorescence Assay and Western Blotting

*P. falciparum* 3D7 strain was cultured with human erythrocytes (4% hematocrit) in RPMI media supplemented with 10% O+ human serum using standard protocol [36]. Thin smears of parasitized red blood cells (RBC) of different developmental stages were prepared and fixed in acetone for 5 minutes followed by chilled methanol for 40 seconds at room temperature. The fixed slides were dried and incubated in 10% fetal calf serum (FCS) in PBS in a humid chamber at 37°C for 2 hour for blocking. The slides were washed with PBS and incubated with purified IgG of anti-PfUDN antibodies (raised in mice) and purified IgG of anti-PfMLH antibodies (raised in rabbit) both at 1∶100 dilutions in PBS containing 10% FCS for 1 hour at 37°C. The slides were then washed four times with PBS for 15 min each and then incubated for 1 hour at 37°C with secondary antibodies (ALEXA 488-green- conjugated- anti-mouse IgG from Invitrogen, USA) and (ALEXA 594-red- conjugated- anti-rabbit IgG from Invitrogen, USA) both diluted 1∶500 in PBS containing 10% FCS. After washing, the slides were incubated in 4′, 6′- di-amidino-2-phenylindole-dihydrochloride (DAPI) for nuclear staining. The slides were washed thrice with PBST (PBS, 0.5% Tween 20) for 10 minutes each and twice with PBS for 10 min each and mounted with antifade reagent Fluroguard purchased from BioRad (Hercules, CA, USA) and viewed under oil immersion. The images were collected using a Bio-Rad 2100 laser-scanning microscope attached to a Nikon 2000U microscope.

For the purpose of western blotting parasites were released from mixed infected erythrocytes cultures by treatment with 0.1% (w/v) saponin. The cell-free protein extracts from early schizont stage parasite cultures were prepared by repeated liquid nitrogen freeze-thaw in the lysis buffer (Pierce, Rockford, IL USA) containing 10 mM Tris–HCl, pH 7.4, 150 mM NaCl, 10 mM EDTA, 1% NP-40, 5% glycerol and protease inhibitor cocktail (Sigma, St. Louis, MO, USA). The extracted proteins were resolved by SDS-PAGE and transferred onto nitrocellulose membrane for the purpose of western blotting using the purified IgG of anti-PfUDN antibodies. The membrane was first incubated overnight at 4°C with a 1∶500 dilution of the purified IgG of anti-PfUDN antibodies and then incubated with the HRP conjugated secondary antibody (Sigma, St. Louis, MO, USA). The blot was developed using Sigma Fast® DAB (3,3′-diaminobenzidine tetrahydrochloride) tablet with urea enhancer tablets (Sigma, St. Louis, MO, USA) according to the manufacturer’s instructions.

**Figure 11:**
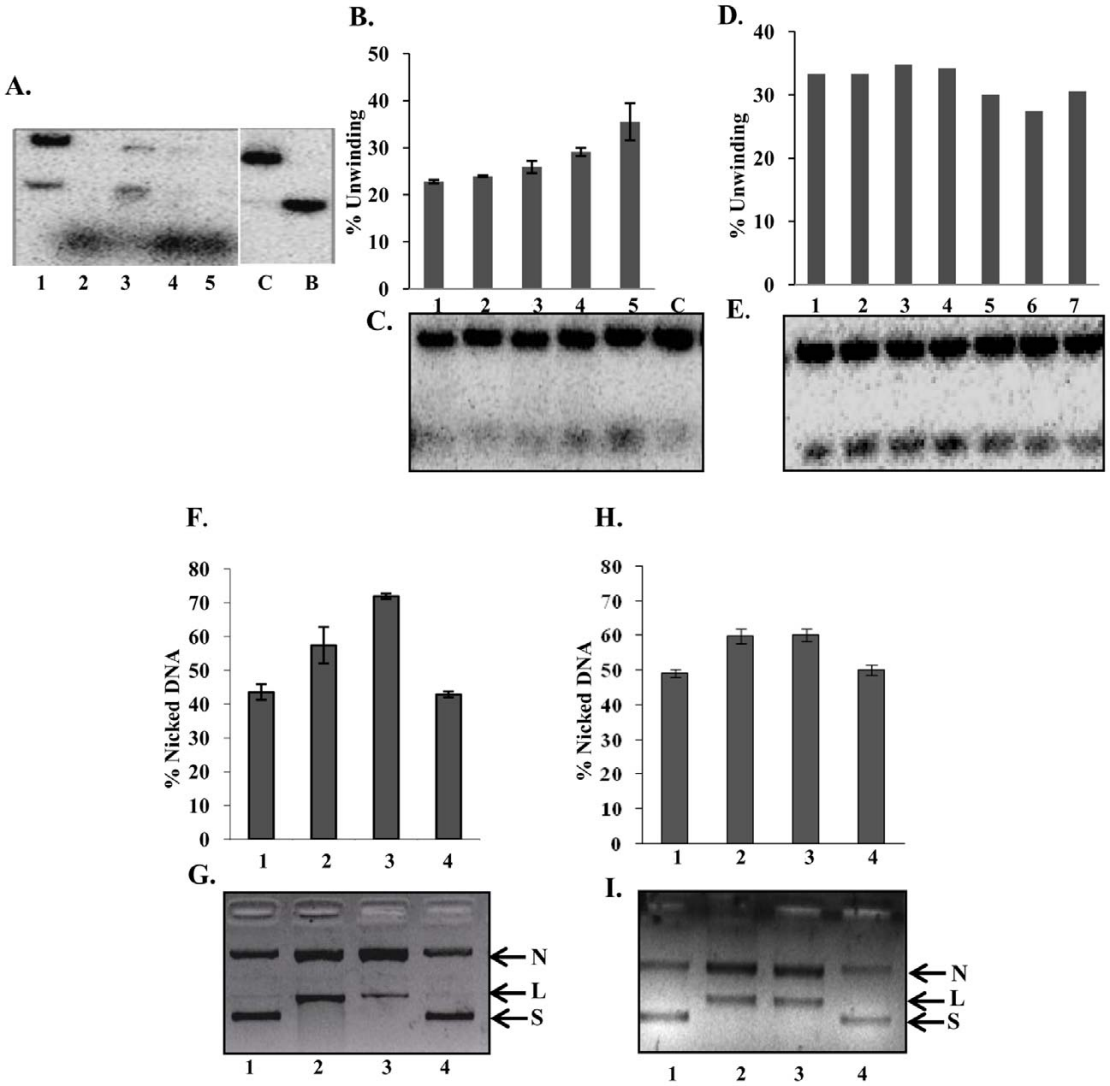
Effect of PfMLH on the helicase activity. **A,** Effect of PfMLH on the helicase activity of purified PfUDN using substrate 1 (Tale S1). Lane 1, 1 µl (10 nM) PfUDN, lane 2, 1 µl PfMLH (8 nM), lane 3, 1 µl PfUDN (10 nM) and 1 µl PfMLH (8 nM), lane 4, 1 µl PfUDN (10 nM) and 2 µl (16 nM) PfMLH, lane 5, 1 µl PfUDN (10 nM) and 3 µl PfMLH (24 nM), lane C, control reaction without any enzyme and lane B is the heat-denatured substrate. **B and C,** Effect of PfMLH on the helicase activity of purified PfUDN using direction-specific substrate (substrate 3B, Tale S1). Lane 1, 1 µl PfUDN (10 nM), lane 2, 1 µl PfUDN (10 nM) and 1 µl PfMLH (8 nM), lane 3, 1 µl PfUDN (10 nM) and 2 µl PfMLH (16 nM), lane 4, 1 µl PfUDN (10 nM) and 1 µl PfMLH (8 nM), lane 5, 1 µl PfUDN (10 nM) and 2 µl PfMLH (16 nM), lane C, control reaction without any enzyme. In lane 3 and 4, PfUDN and PfMLH were added at the same time in the substrate. In lane 5 and lane 6, the substrate mixture was pre-incubated with 1 and 2 µl of PfMLH for 10 minutes before the addition of PfUDN. The amount of unwound DNA was quantitated and plotted as a histogram above the gel picture. **D,** Effect of heat treated PfMLH, PfMLHN and PfMLHC on the helicase activity of purified PfUDN using direction-specific substrate (substrate 3B, Tale S1). Lane 1, 1.5 µl PfUDN (15 nM), lane 2, 1.5 µl PfUDN (15 nM) and 1 µl heat treated PfMLH (8 nM), lane 3, 1.5 µl PfUDN (15 nM) and 1 µl PfMLHN (8 nM), lane 4, 1.5 µl PfUDN (15 nM) and 1 µl PfMLHC (8 nM), lane 5, 1.5 µl PfUDN (15 nM) and 1 µl heat treated PfMLH (8 nM), lane 6, 1.5 µl PfUDN (15 nM) and 1 µl PfMLHN (8 nM), and lane 7, 1.5 µl PfUDN (15 nM) and 1 µl PfMLHC (8 nM). In lanes 2, 3 and 4, PfUDN and heat treated PfMLH, PfMLHN and PfMLHC were added at the same time in the substrate. In lane 5, 6 and 7, the substrate mixture was pre-incubated with 1 µl of heat treated PfMLH, PfMLHN and PfMLHC for 10 minutes before the addition of PfUDN. **E,** The amount of unwound DNA was quantitated and plotted as a histogram above the gel picture. **F and G,** Effect of PfUDN on the endonuclease activity of purified PfMLH. Lane 1, negative control reaction without enzyme, lane 2, reaction with purified PfMLH (20 nM), lane 3, reaction with purified PfMLH (20 nM) and PfUDN (15 nM) and lane 4, reaction with purified PfUDN (15 nM). **H and I,** Effect of PfUDC1 on the endonuclease activity of purified PfMLH. Lane 1, negative control reaction without enzyme, lane 2, reaction with purified PfMLH (20 nM), lane 3, reaction with purified PfMLH (20 nM) and PfUDC1 (15 nM) and lane 4, reaction with purified PfUDC1 (15 nM). The arrows show the position of nicked circular (N), linear (L) and supercoiled (S) plasmid DNA. The percent amount of nicked DNA was quantitated and plotted as a bar diagram above the gel picture. Each bar represents the mean percentage ± SD.

## Results

### Identification and Sequence Analysis of PfUvrD

UvrD is a member of superfamily 1 of helicases. An alignment of the complete amino-acid sequence of UvrD homologue from *P. falciparum* with *E. coli* UvrD using BLAST (http://blast.ncbi.nlm.nih.gov/Blast) revealed that PfUvrD aligned contiguously with its *E. coli* counterpart (Figure 1). Further detailed analysis of the protein sequence at Expasy (http://prosite.expasy.org) indicated that similar to *E. coli* UvrD, PfUvrD also contains two distinct domains: an UvrD like DNA helicase ATP-binding domain and an UvrD like DNA helicase C-terminal domain (Figure 2A and 2B). It contains Q motif at the extreme N-terminal and all the conserved motifs from Ia-Id, II, III, IV, IVa-IVc, V, Va, VI and VIa (Figure 1, Figure 2C) [28]. Similar to UvrD from other sources PfUvrD also contains all the motifs clustered in its N-terminal region (Figure 2C) [37]. A detailed analysis of PfUvrD amino acid sequence indicated that the distance between each motif is variable between *E. coli* and PfUvrD proteins [22] (Figure 1, Figure 2C). As reported earlier *E. coli* UvrD contains four domains (1A, 1B, 2A and 2B) and a C-terminal extension [29]. A comparison of PfUvrD sequence with *E. coli* UvrD shows that it also contains all these domains but no C-terminal extension (Figure 1). The 1A domain in PfUvrD is from amino acid 1–722 and the 1B domain is from amino acid 150–464 (Figure 1, orange and light blue boxes respectively). The 2A domain in PfUvrD is from amino acid 723–1441 and the 2B domain is from amino acid 896–1359 (Figure 1, purple and green boxes respectively). The PlasmoDB number for PfUvrD is PFE0705c. The blast analysis of PfUvrD against PlasmoDB database (www.plasmodb.org) revealed that this gene is located on chromosome 5 of *P. falciparum* and it contains no introns.

**Figure 12:**
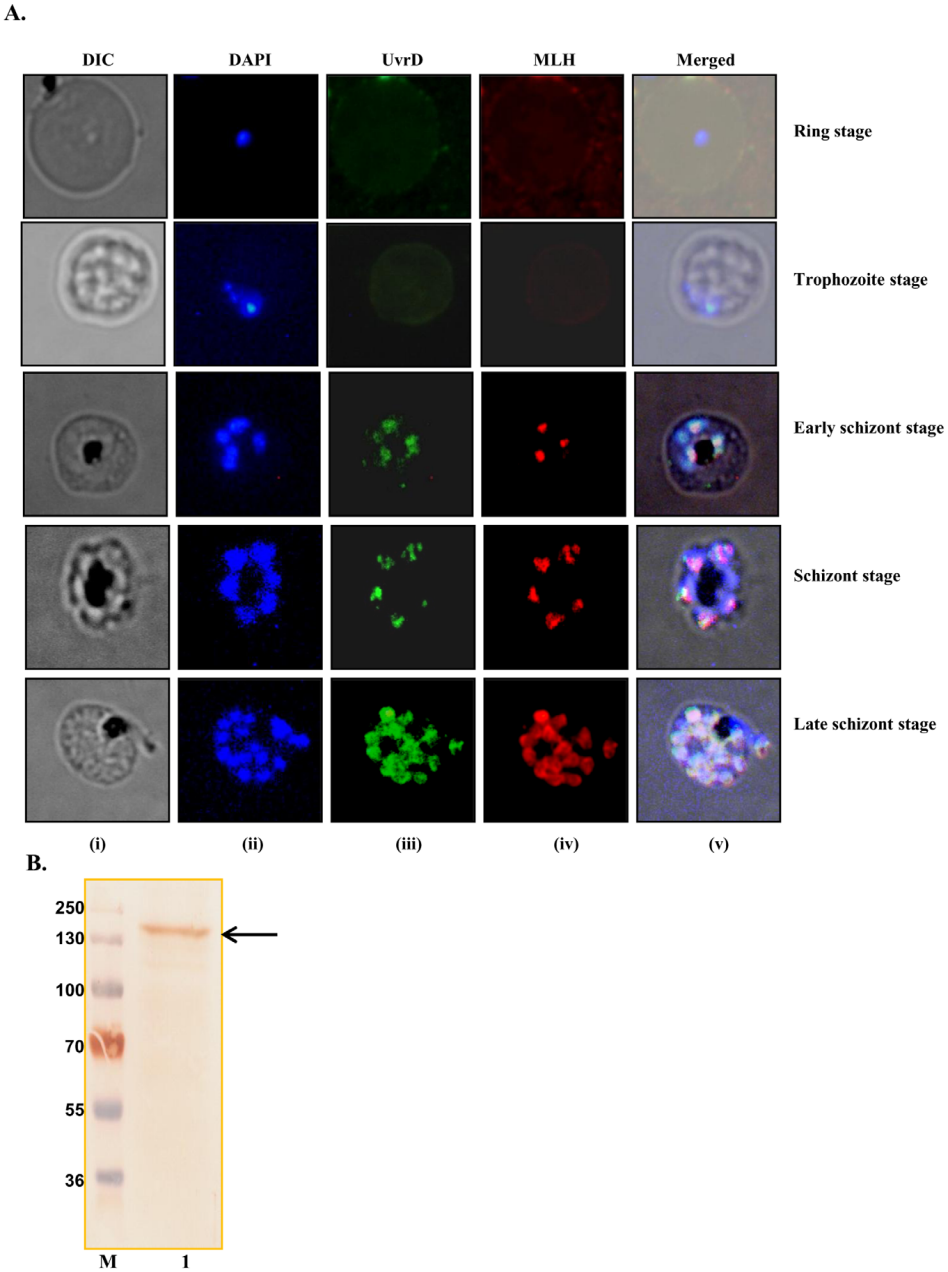
Localization of PfUvrD. **A,** Immunofluorescence staining. The cells were fixed and immunostained. Panel (i) phase (ii) image of cell stained with DAPI (iii) immunofluorescently stained cell (green, *P. falciparum* UvrD) (iv) immunofluorescently stained cell (red, *P. falciparum* MLH) and (v) super-imposed images. Control normal mouse sera produced no fluorescence (data not shown). **B,** Western blot analysis. Lane M is the protein molecular weight marker and lane 1 is protein from early schizont stages of the intraerythrocytic developmental of the parasite. The arrow shows the PfUvrD band.

The nucleotide sequence of PfUvrD is 4326 bases and it codes for a protein of 1441 amino acids. For the amplification of full-length PfUvrD, genomic DNA from *P. falciparum* 3D7 strain was used with the primer pair PfUF1 and PfUR3. On repeated trials, we were not able to obtain the amplification of the full-length PfUvrD from genomic DNA or cDNA preparations. Therefore the sequence was divided according to the presence of different domains as shown into N terminal, PfUDN (2079 bases, ∼82 kDa) containing UvrD helicase domain (domain 1A and 1B) [29], which consists of motifs Q, Ia-Id, II, III and part of motif IV (Figure 2D), and C terminal 1, PfUDC1 (1128 bases, ∼45 kDa) containing UvrD helicase C terminal domain (first half of domain 2A and 2B), which consists of remaining part of motif IV and motifs IVa–IVc and 161 amino acids of intervening sequence between motif IV and V (Figure 2E). The C terminal 2, PfUDC2 (1020 bases, ∼40 kDa, consisting of second half of domain 2A and 2B) contains remaining part (261 amino acids) of intervening sequence between motif IV and V and motifs V, Va, VI and VIa (Figure 2F). Each fragment i.e. PfUDN, PfUDC1 and PfUDC2 was amplified and cloned as described in materials and methods.

### Molecular Modeling of PfUvrD Structure2

For structural modeling the sequence of full-length PfUvrD was submitted to the Swissmodel homology-modeling server (http://swissmodel.expasy.org/) [38]. A total of five models were obtained and four models covered the areas ranging from 57 to 168 amino acids of PfUvrD only but only one model covered a larger range (amino acid 32–815) of the PfUvrD sequence (Data S1). Therefore this model which was built using PcrA DNA helicase from *B. stearothermophilus* as template was studied in detail [27]. PfUvrD primary sequence residues 32 to 815 showed ∼13% identity to the PcrA DNA helicase from *B. stearothermophilus* [27]. The structural modeling of the PfUvrD was therefore done using the known crystal structure of this homologue as the template (PDB number 3pjrA at http://www.ncbi.nlm.nih.gov/Structure/mmdb). The ribbon diagram of the template is shown in Figure 2G and the predicted structure of PfUvrD is shown in Figure 2H. When the modeled structure of PfUvrD and the template were superimposed, it is clear that these structures superimpose partially (Figure 2I). Molecular graphic images were produced using the UCSF Chimera package (http://www.cgl.ucsf.edu/chimera) from the Resource for Biocomputing, Visualization, and Informatics at the University of California, San Francisco (supported by NIH P41 RR-01081) [39]. Further structure analysis with chimera using matchmaker structure comparison tool revealed that the RMSD between 303 atom pairs is 0.295 angstroms [40]. The RMSD value corresponds to the amino acid residue pairs which align perfectly in the pairwise alignment (Data S1).

### Purification of PfUvrD Fragments and Characterization of ATPase and DNA Helicase Activities

The expression clones corresponding to each fragment such as PfUDN, PfUDC1 and PfUDC2 were transformed into *E. coli* strain BL21 (DE3) pLysS and the recombinant proteins were purified using method described in materials and methods section. The SDS–PAGE analysis followed by silver staining of the purified proteins showed that all the fragments PfUDN (Figure 3A, lanes 1 and 2), PfUDC1 (Figure 3B, lane 1) and PfUDC2 (Figure 3C, lane 1) contain almost no contaminating protein and are homogeneous preparations. The purified fractions were further checked by western blot analysis using anti-His antibodies and only a single band in each of the purified fraction was detected for PfUDN (Figure 3D, lanes 1 and 2), PfUDC1 (Figure 3E, lane 1) and PfUDC2 (Figure 3F, lane 1) respectively. These purified preparations were used for all of the assays described in the following sections. The purified PfUDN protein was also used for the production of polyclonal antibodies in mice.

The ssDNA-dependent ATPase activity of PfUDN, PfUDC1 and PfUDC2 was checked using standard assay conditions as described in materials and methods in the presence of traces of radiolabelled ATP with 1 mM cold ATP and purified enzymes. The concentration-dependence of ATPase activity was checked by using 6 to 72 nM of PfUDN and 20 to 260 nM of PfUDC1 proteins. The percent release of radioactive inorganic phosphate (Pi) from [γ^32^P] ATP was measured. The results clearly showed that PfUDN (Figure 3G and 3H, lanes 1–4) and PfUDC1 contain concentration and ssDNA dependent ATPase activity (Figure 3K and 3L, lanes 1–4). The ATPase reaction using 72 nM of purified PfUDN and 40 nM of purified PfUDC1 at different time points was carried out in order to study the time dependence of ATPase activity. The percent release of radioactive Pi from [γ-^32^P] ATP showed linearity up to 60 minutes in both PfUDN and PfUDC1 (Figure 3I and 3J, lanes 1–5 and Figure 3M and 3N, lanes 1–5). On repeated trials we were unable to detect any ATPase activity in PfUDC2 (data not shown).

The standard helicase strand-displacement assay measures the unwinding of ^32^P-labelled DNA fragment from a partially duplex nucleic acid. In order to characterize the DNA unwinding activity of PfUDN, PfUDC1 and PfUDC2 the standard strand-displacement assay was used. The DNA unwinding activity using different concentration of purified PfUDN (6 to 72 nM) and PfUDC1 (45 to 275 nM) and optimal assay conditions as described in materials and methods with ∼1000 cpm of the substrate 1 (Table S1) in buffer having 1 mM ATP, 1 mM MgCl_2_ and 75 mM KCl was tested. It is interesting to note that PfUDN (Figure 4A and 4B, lanes 1–7) and PfUDC1 (Figure 4C and 4D, lanes 1–4) both showed the concentration-dependent helicase activity. On repeated trials we were unable to detect any helicase activity in PfUDC2 (Figure 4E, lanes 1–3).

**Figure 13:**
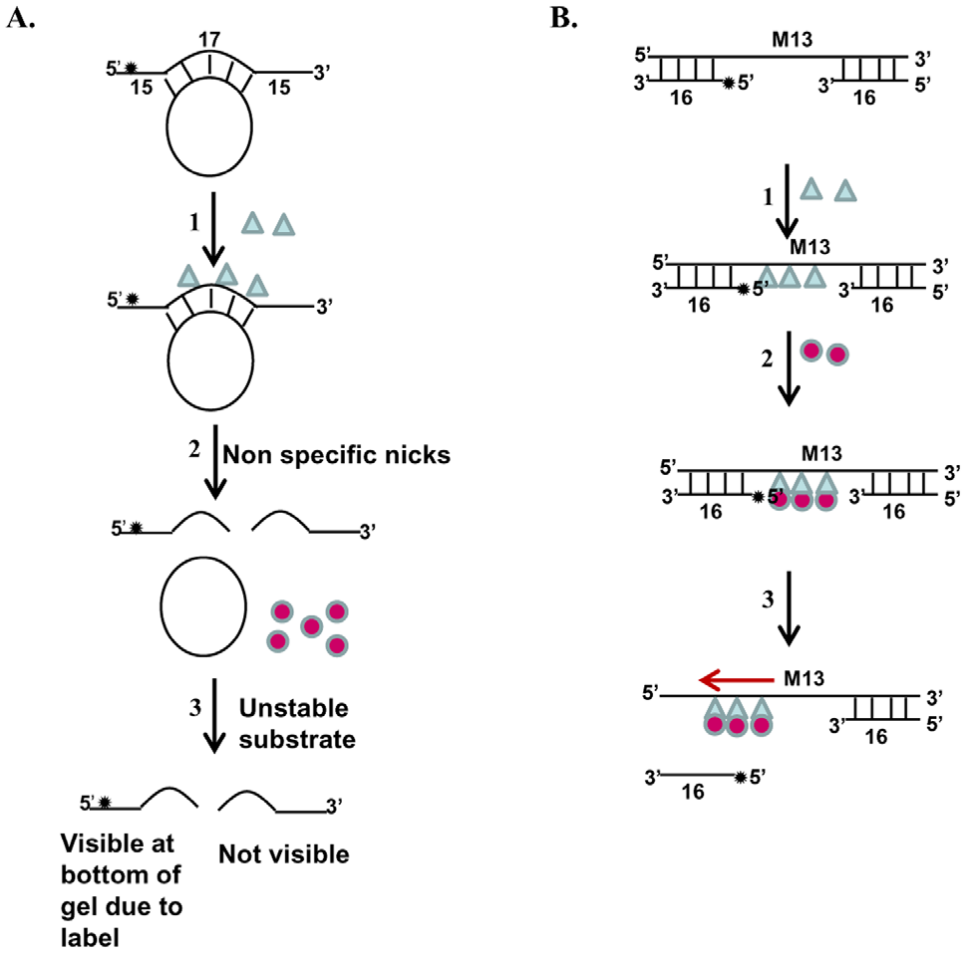
Models for PfMLH-stimulated unwinding. A, Using normal partially duplex circular substrate (substrate 1, Tale S1). 1. Incubation with PfMLH. 2. It creates non-specific nicks. 3. It results in unstable substrate, which melts at 37°C and addition of PfUDN has no effect. **B,** Using direction-specific substrate (substrate 3B, Tale S1). 1. Incubation with PfMLH results in its binding to substrate. 2. Incubation with PfUDN facilitates its loading to the substrate through PfMLH. 3. It results in stimulation of unwinding in 3′ to 5′ direction.

### Immunodepletion of ATPase and Helicase Activities of PfUDN and PfUDC1

Purified PfUDN was allowed to react separately with IgGs purified from the pre-immune sera and from the sera of the mice immunized with PfUDN using the method described in materials and methods section. We were unable to generate antibodies against purified PfUDC1 therefore for this assay purified PfUDC1 was allowed to react with anti-His antibodies. The immunodepleted supernatants were checked for various activities. The results showed that the ATPase activity of PfUDN (Figure 5A, lanes 1–3) and PfUDC1 (Figure 5B, lanes 1–3) was depleted with the specific or anti-His antibodies. On the contrary the samples treated with pre-immune IgG of both PfUDN and PfUDC1 showed concentration-dependent ATPase activity (Figure 5A, lanes 4–6 and Figure 5B, lanes 4–6 respectively). Similar results were obtained with helicase activity also. The results showed that the helicase activity of PfUDN using substrate 1 (Table S1) (Figure 5C, lanes 1–3) and PfUDC1 (Figure 5D, lanes 1–3) was also depleted with the specific or anti-His antibodies. But the samples treated with pre-immune IgG of both PfUDN and PfUDC1 showed concentration-dependent helicase activity (Figure 5C, lanes 4–6 and Figure 5D, lanes 4–6 respectively). These data further confirm that the ATPase and helicase activities are due to the purified PfUDN and PfUDC1 proteins and not due to any contamination in the purified preparations. This experiment was repeated with two different preparations of PfUDN and PfUDC1 proteins and each bar in Figure 5A–D represents the mean percentage ± SD of two different experiments.

### Determination of *K_m_* and *V_max_* for the Helicase Activity of PfUDN and PfUDC1

Helicase assay reactions were performed using the substrate 1 (Table S1) of different concentrations (5–40 nM) in a standard reaction buffer. The amount of dsDNA and unwound ssDNA was quantified as described in materials and methods section. A conventional hyperbolic dependence of the rate of reaction on substrate concentration was obtained, such that the rate of substrate unwinding was initially linear and later saturated with increasing substrate concentrations that gave best-fit to the Michaelis–Menten equation. The K_m_ and V_max_ of helicase activity for PfUDN and PfUDC1 was measured by using Sigma plot software (http://www.sigmaplot.com/). Nonlinear regression analysis of this data yielded a *Km* value of 1.2±0.1 nM and 3.2±0.4 nM for PfUDN (Figure 6A) and PfUDC1 respectively (Figure 6B). The *Vmax* value is 0.1920 nM/min/ng and 0.4048 nM/min/ng for PfUDN and PfUDC1 respectively.

### Further Characterization of Unwinding Activity

Further characterization of unwinding activity was done using PfUDN only. It is well established that helicases have specific nucleotide requirement to couple the hydrolysis of nucleotide to unwinding activity. Therefore the helicase activity of PfUDN was measured with different deoxynucleotide triphosphates and nucleotide triphosphates using substrate 1 (Tale S1). It is interesting to note that PfUDN showed the unwinding activity in the presence of all the dNTPs and NTPs such as dCTP, CTP, dGTP, GTP, dATP, ATP, dTTP and UTP used for the reaction (Figure 7A and 7B, lanes 1–8 respectively). On the other hand there was no unwinding activity of PfUDN in the absence of any NTP or dNTP (Figure 7A, lane E). The concentration requirement using ATP showed that the unwinding activity of PfUDN was maximal at 2.5 mM ATP concentration and it did not increase further on increasing the ATP concentration to 5.0 mM (Figure 7C and 7D, lane 5 and 6 respectively). It is interesting to note that PfUDN did not show any unwinding activity in the absence of ATP (Figure 7C, lane E).

In order to determine the specificity of PfUDN, its DNA unwinding activity was tested with blunt end duplex substrate also (substrate 2, Tale S1). This substrate had blunt ends but contained identical core sequence and same duplex length (17 basepair) so that as far as possible, any differences in efficiency of unwinding due to sequence differences could be eliminated. The assay was performed using the method described in the previous section. The results clearly indicate that PfUDN unwinds the blunt end duplex substrate (substrate 2, Tale S1) also in concentration-dependent manner (Figure 7E and 7F, lanes 1–6).

### Determination of Direction of Unwinding by PfUDN

It is well established that all the helicases preferentially unwind nucleic acids in a polar fashion by moving unidirectionally on the bound strand in a duplex. The direction of unwinding by a helicase is defined by the strand to which the enzyme binds and moves. The unwinding activity of purified PfUDN was tested by using two different direction-specific substrates, one specific for the 5′ to 3′ (substrate 3A, Tale S1) and the other for the 3′ to 5′ (substrate 3B, Tale S1) direction, prepared as described in materials and methods section. The DNA unwinding activity, using both the direction-specific substrates with different concentrations of PfUDN enzyme was determined. The release of radiolabelled DNA from the substrates of Figure 8A and 8C by PfUDN enzyme will indicate the movement in 5′ to 3′ and 3′ to 5′ directions, respectively. The results show that PfUDN was unable to show the activity with the 5′ to 3′ direction-specific substrate (substrate 3A, Tale S1) (Figure 8A, lanes 1–6). On the other hand it is evident from the results that PfUDN could unwind the 3′ to 5′ direction-specific duplex substrate (substrate 3B, Tale S1) very efficiently (Figure 8D, lanes 1–6) indicating that it contains unidirectional DNA unwinding activity. The helicase activity with this substrate was also directly proportional to the concentration of PfUDN used in the reaction (Figure 8D and 8E, lanes 1–6). It should be noted here that the 72 nM of PfUDN resulted in complete unwinding of the direction-specific substrate (substrate 3B, Tale S1) (Figure 8D, lane 6) as opposed to ∼30% unwinding observed with the normal substrate (substrate 1, Tale S1) (Figure 4A, lane 7). This difference in activity may be due to the difference in the processivity of the PfUDN.

### Purification of Endogenous UvrD Protein from *P. falciparum* and its Activity Analysis

The purification of endogenous *P. falciparum* 3D7 UvrD protein was performed as described in materials and methods section. The standard protocol was used with slight modification mentioned in the material and methods section. Before performing the immunoprecipitation, the parasite lysate was first precleared with control agarose resin then with pre-immune IgG-protein A-Sepharose column to reduce non specific protein binding. The purified IgGs from pre-immune serum and anti-PfUDN serum were cross-linked successfully and to minimize any non specific binding, both the columns were washed stringently with the lysis buffer. To remove the detergent, the columns were washed three times with the wash buffer (1X TBS). Both column elutes (preimmune column and anti-PfUDN column elute) were checked with SDS-PAGE coupled western blot analysis and the results clearly indicate the presence of PfUvrD of ∼170 kDa in the anti-PfUDN column elute (Figure 9A, lane 1). Traces of heavy chain of IgG of secondary anti mouse antibody were also coeluted/detected in both the anti-PfUDN column elute as well as the preimmune column elute (Figure 9A, lanes 1 and 2 respectively).

Different concentrations of the recovered endogenous PfUvrD protein were used for the helicase activity assay using the substrate 1 (Tale S1) and the method described. We also used the pre immune elute as a control for the enzymatic assays. The results reveal that the *P. falciparum* endogenous UvrD protein contains concentration-dependent DNA helicase activity (Figure 9B and 9C, lanes 5–8). The elute obtained with pre-immune serum contained some background activity (Figure 9B and 9C, lanes 1–4).

### Further Characterization of Unwinding Activity of Endogenous PfUvrD

Further characterization of unwinding activity of endogenous PfUvrD was also done. The helicase activity of endogenous PfUvrD was also measured with different deoxynucleotide triphosphates and nucleotide triphosphates using substrate 1 (Tale S1). It is interesting to note that similar to PfUDN, endogenous PfUvrD also showed the unwinding activity in the presence of all the dNTPs and NTPs such as dCTP, CTP, dGTP, GTP, dATP, ATP, dTTP and UTP used for the reaction (Figure 9D and 9E, lanes 1–8 respectively). Similar to PfUDN, endogenous PfUvrD also did not show any unwinding activity in the absence of any NTP or dNTP (Figure 9D, lane E).

### Interaction of PfUvrD Fragments with PfMLH

The functions of most of the proteins are dependent upon their direct physical interactions with other polypeptides within a cell. It has been shown previously that *E. coli* MutL and UvrD proteins interact [41]. In a recent study from our laboratory we have reported the identification and functional characterization of PfMLH from *P. falciparum* where we reported that PfMLH is a component of MMR and it contains weak ATPase and endonuclease activity [31]. In the present study using ELISA based assays we performed the interaction analysis as described in materials and methods section. PfUDN, PfUDC1, PfUDC2 and PfMLH were used for the interaction study. The results show that only PfUDN interacts with PfMLH (Figure 10A) and PfUDC1 and PfUDC2 had no detectable interaction with PfMLH (Figure 10A). Furthermore the results suggest that this interaction is concentration-dependent (Figure 10A).

### In vitro Protein-protein Interaction Study Using Immunoaffinity Column

The in vitro protein-protein interaction study was done using preimmune and anti-PfUDN immunoaffinity column as described in materials and methods section. Equal amount of (PfUDN and PfMLH) mixture was incubated with protein A sepharose coupled to preimmune or anti-PfUDN IgGs. Both the column elutes were separated by SDS PAGE coupled western blot analysis. The results clearly show that in addition to PfUDN (Figure 10B, lane 1), PfMLH (Figure 10B, lane 2) was also detected in the eluted fractions of the anti-PfUDN column because it interacted with PfUDN. On the other hand PfMLH was not detected in the eluted fractions of the anti-preimmune column (Figure 10B, lane 3). These results further confirm the interaction between PfUDN and PfMLH proteins.

### Helicase Assays in the Presence of PfMLH

We have reported previously that PfMLH contains no helicase activity [31]. To investigate the effect of PfMLH on the activity of PfUDN, the helicase activity of PfUDN was assayed in the presence of differing amounts of purified PfMLH protein [31]. Using the substrate 1 (Tale S1) we were able to show that PfUDN contains unwinding activity (Figure 11A, lane 1). But using this substrate we were unable to observe the stimulation of unwinding activity. Because it is most likely that before the helicase PfUDN could reach the duplex area in this normal circular substrate for unwinding from the 3′ end, the substrate was nicked and degraded due to the non-specific nicking endonuclease activity of PfMLH. Due to this degradation it was not possible to observe the effect of PfMLH on the unwinding activity as is evident from the results shown (Figure 11A, lanes 3–5, increasing concentration of PfMLH with constant concentration of PfUDN).

Therefore in order to check the effect of PfMLH on unwinding activity we decided to use the 3′ to 5′ direction-specific substrate (substrate 3B, Tale S1). The assay conditions were selected so that the PfUDN (10 nM) alone unwound ∼25% of the helicase substrate in 60 minutes as described under materials and methods section. After 60 minutes of incubation the reactions were stopped and the products were analyzed by gel electrophoresis and visualized by autoradiography. It is interesting to note that PfMLH stimulated the unwinding activity of PfUDN in a concentration-dependent manner only when the substrate mixture was pre-incubated with PfMLH prior to the addition of PfUDN. This stimulation was concentration-dependent and ∼22–36% stimulation was observed at the two concentrations of PfMLH added as compared to the activity obtained by PfUDN alone (Figure 11B and 11C, lane 1 versus lane 4 and 5 respectively). The minimum concentration of PfMLH for the stimulatory effect of PfUDN helicase activity was 8 nM. We were unable to detect the stimulation of the helicase activity below this concentration**.** On the other hand when PfUDN and two concentrations of PfMLH were added together in the substrate mixture the stimulation of activity was almost negligible (Figure 11B and 11C, lane 1 versus lanes 2 and 3). As reported earlier also PfMLH alone had no detectable helicase activity [31]. This experiment was also repeated at least three times and the results were reproducible. To further confirm that the stimulation of helicase activity is due to the PfMLH, we also used the catalytically inactive N-terminal (PfMLHN), C-terminal (PfMLHC) [31] and the heat inactivated PfMLH (which loses its activity) in the helicase reaction. This experiment was also done using the direction-specific substrate (substrate 3B, Tale S1). The results clearly indicate that there was no significant effect on the helicase activity of PfUDN (12 nM) (Figure 11D and 11E, lane 1) when the heat treated PfMLH (8 nM), PfMLHN (8 nM) or PfMLHC (8 nM) were used (Figure 11D and 11E, lanes 2, 3 and 4 respectively). Furthermore when the heat treated PfMLH (8 nM), PfMLHN (8 nM) or PfMLHC (8 nM) were pre-incubated prior to the addition of PfUDN (12 nM), there was still no effect but a slight inhibition of unwinding activity was observed (Figure 11D and 11E, lane 1 versus lanes 5, 6 and 7 respectively).

### Endonuclease Assays in the Presence of PfUDN

As reported in previous sections PfUDN and PfMLH interact and there is no detectable interaction between PfMLH, PfUDC1 and PfUDC2. In a previous study we have reported that PfMLH contains endonuclease activity [31]. Therefore we investigated the effect of PfUDN and PfUDC1 on the endonuclease activity of PfMLH. To check this effect of PfUDN and PfUDC1 on endonuclease activity, the endonuclease assay was performed in the presence and absence of PfMLH using the method described in the material and methods section. The endonuclease activity was checked by using supercoiled pBR322 DNA, PfMLH (20 nM), PfUDN (15 nM) and/or PfUDC1 (15 nM) proteins. The assay of PfMLH with supercoiled DNA resulted into the nicking of supercoiled pBR322 (Figure 11F and 11G, lane 2) as compared to no protein control (Figure 11F and 11G, lane 1). The results further indicated that the endonuclease activity of PfMLH (20 nM) is modulated significantly in the presence of PfUDN (15 nM) (Figure 11F and 11G, lane 3). On the other hand PfUDN (15 nM) alone had no detectable endonuclease activity (Figure 11F and 11G, lane 4). Further interpretations of this result is interesting that in the presence of PfUDN (15 nM), the percentage of the nicked DNA increased significantly from ∼56% to ∼75% while percentage of linear DNA decreased in the same ratio. On the other hand PfUDC1 had no effect on the endonuclease activity of PfMLH. The reaction of PfMLH (20 nM) with supercoiled DNA resulted in nicking of supercoiled DNA (Figure 11H and 11I, lane 2) as compared to control without protein (Figure 11H and 11I, lane 1) but the addition of PfUDC1 (15 nM) had almost no effect on the endonuclease activity of PfMLH (Figure 11H and 11I, lane 3). PfUDC1 (15 nM) alone contains no detectable endonuclease activity (Figure 11H and 11I, lane 4).

### Localization of PfUvrD by Immunofluorescence Assay and Western Blotting

The purified antibodies to PfMLH and PfUDN were used to study the localization of these proteins in *P. falciparum* by immunohistochemical methods. To localize PfMLH and PfUvrD, we also performed co-localization studies. We have reported in a recent study that there was no detectable expression of PfMLH in the ring and trophozoite stages and the expression was mainly observed in the schizont stages of the development of the parasite [31]. In the case of PfUvrD also, there was no detectable expression in the ring and trophozoite stages (Figure 12A). Therefore the co-localization studies were done using three different stages of intraerythrocytic development such as early schizont, schizont and late schizont stages of the parasite. The results suggest that PfUvrD is mainly localized in the nucleus and further indicate that most likely PfUvrD is also expressed in a cell-cycle dependent manner with peak expression in the schizont stages of the development (Figure 12A). The nuclei were identified by DAPI staining and the overlay of all the images is shown in Figure 12A (v). The results further show that both of these proteins co-localize in all the stages of the development of the parasite (Figure 12A). The purified antibodies were also used to detect the level of expression of PfUvrD in the 3D7 parasite lysate. The purified IgG recognized only a single protein of right size (∼170 kDa) in the lysate prepared from early schizont stages of intraerythrocytic developmental cycle of malaria parasite-infected RBCs (Figure 12B, lane 1).

## Discussion

In this study we have characterized the *P. falciparum* UvrD homologue in detail. The size of PfUvrD (1441 amino acid, ∼170 kDa) is almost two times as compared to *E. coli* UvrD (720 amino acid, ∼82 kDa). The activity analysis showed that the helicase and ssDNA-dependent activities are present in the N-terminal PfUDN and the C-terminal first half PfUDC1 fragments. It is interesting to note that PfUDN, termed as UvrD helicase ATP binding region (containing domain 1A and 1B) [28], consists of motifs Q, Ia-Id, II, III and part of motif IV only but it shows the ATP hydrolyzing and unwinding activities. PfUDC1 termed as UvrD helicase C terminal region (containing first half of domain 2A and 2B) [28], which consists of remaining part of motif IV and motifs IVa-IVc and 161 amino acids of intervening sequence between motif IV and V also contains both ATP hydrolyzing and unwinding activities. To the best of our knowledge this is the first report showing both the enzyme activities individually in the N and C terminal fragments of the protein UvrD. On the other hand the PfUDC2 domain, which contains the second half of domain 2A and 2B and remaining 257 amino acids of the intervening sequence between motif IV and V in addition to motifs V, Va, VI and VIa did not show any enzyme activity. In previous studies it has been reported that UvrD contains four structural domains 1A, 1B, 2A and 2B and domains 1A and 2A form the core of the helicase [28], [29]. Our results presented here suggest that PfUDN which has only domain 1A and 1B contains ATPase activity and is capable of unwinding the duplex DNA. Similarly PfUDC2 which has only first half of domain 2A and 2B also contains the ATPase and helicase activities. The *E. coli* Rep protein which is structurally homologous to *E. coli* UvrD protein has been characterized and it was reported that the 2B subdomain of the *E. coli* Rep protein is not required for DNA helicase activity in vitro [42]. The results presented here support these findings and further suggest that in PfUDN, only domain 1A and 1B are enough for helicase activity. On the other hand in PfUDC1, only the first half of domain 2A and 2B are sufficient for helicase activity. The characterization of *E. coli* UvrD_1–647_, in which the entire C-terminal extension was removed but domain 2A was not disrupted showed that UvrD_1–647_ is an active ATPase and retains helicase activity on a wide variety of substrates [29].

Our results suggest that the optimum concentration requirement of ATP for the unwinding activity of PfUDN is 2.5 mM. It has been reported previously that *Mycobacterium tuberculosis* UvrD helicase showed ∼80% unwinding activity at 1 mM ATP concentration [37]. It is noteworthy that PfUDN utilized all the NTPs or dNTPs as cofactor for the unwinding activity. These results are contrary to the nucleotide requirement reported for *M. tuberculosis* UvrD helicase, which can use only ATP and dATP as cofactor for its unwinding activity [37]. The SRS2 DNA helicase of yeast is a homologue of bacterial UvrD helicase and it can also use only ATP or dATP as cofactor for its unwinding activity [43]. It has been reported previously that *Thermus thermophilus* UvrD helicase activity was supported by ATP and dATP and up to 40%–50% by GTP and dGTP but other nucleotides like CTP, dCTP, UTP and dTTP supported the activity weakly or not at all [44]. In a recent study it has been reported that *H. pylori* (Hp) UvrD was able to hydrolyze and utilize GTP for its helicase activity although not as effectively as ATP [25]. Our results suggest that PfUDN can efficiently unwind the blunt end duplex substrate also. These results are in contrast to the *Haemophilus influenza* and HpUvrD, which required a minimum of 12 and 20 or more nucleotides of 3′-single-stranded DNA tail respectively for efficient unwinding of duplex DNA [25]. *M. tuberculosis* UvrD helicase also required a 3′ single-stranded DNA tail of 18 nucleotides for efficient unwinding [37]. On the other hand it was reported that *E. coli* UvrD could unwind fully duplex blunt ended DNA substrates [45]. The unwinding of blunt end duplex by PfUDN might help it to work on wide spectra of nucleic acid targets in the parasite. Most of the helicases belonging to PUR (Pcr/UvrD/Rep) family contain 3′ to 5′ polarity of unwinding. The direction of unwinding by PfUDN is 3′ to 5′, which is similar to the polarity reported for *M. tuberculosis* UvrD helicase [37]. *Thermoanaerobacter tengcongensis* UvrD also showed a 3′ to 5′ polarity of unwinding [46]. On the other hand PcrA from *Bacillus anthracis* showed robust bidirectional 3′ to 5′ as well as 5′ to 3′ helicase activities [47]. Our results show that the endogenous PfUvrD contains DNA helicase activity and its characteristics are similar to the helicase activity observed by PfUDN. To the best of our knowledge this is the second report demonstrating the helicase activity of an endogenous *P. falciparum* protein. Recently using similar approaches we reported the enzymatic activities of an endogenous PfRuvB3 family of protein [48].

Using two different assays we have shown that PfUDN and PfMLH interact with each other, while PfUDC1 and PfUDC2 show no detectable interaction with PfMLH. As a result of this interaction the unwinding activity of PfUDN was stimulated in the presence of PfMLH to some extent. These data are in agreement with the previously reported data for *T. tengcongensis* UvrD helicase [46]. The extent of stimulation of helicase activity by MutL varies significantly in different systems. The stimulation of unwinding activity of *T. tengcongensis* UvrD by MutL was reported to be ∼30% [46]. On the other hand the stimulation of helicase activity of *E. coli* UvrD by MutL is about 10 fold [20], [49]. It is interesting to note that we were able to observe the effect of PfMLH on the activity of PfUDN when the direction specific substrate was used because PfUDN has to bind and move in single direction only. Similar results have been reported previously also where in the case of Aae (*Aquifex aeolicus*) MutL and the putative *A. aeolicus* DNA helicase (Aq793) the enhancement was observed by using 3′ overhang substrate only [50]. Based on our observation we have proposed a model for the detection of stimulation of unwinding using the direction-specific substrate only (Figure 13). Our experimental data suggest that PfMLH binds and nicks a normal partially duplex circular substrate resulting in formation of shorter duplex, which is unstable at 37°C and melts (Figure 11A, lane 2, Figure 13A, steps 2 and 3). Further addition of PfUDN results in no unwinding due to depletion of substrate (Figure 11A, lanes 3–5, and Figure 13A, step 3). On the other hand using the direction-specific substrate, most likely PfMLH first binds to the substrate and helps in the loading of PfUDN on its addition, which results in stimulation of unwinding (Figure 11B and 11C, lane 1, lanes 4–5, Figure 13B, steps 1–3).

Overall in the present study we have reported that the helicase and ssDNA-dependent ATPase activities are present in the N-terminal and first half of the C terminal of *P. falciparum* UvrD proteins. We have also demonstrated that the endogenous PfUvrD contains the characteristic DNA helicase activity. PfMLH interacts with PfUDN and positively regulates its helicase activity and PfUDN stimulates the endonuclease activity of PfMLH. PfUvrD protein is expressed in the schizont stages of intraerythrocytic development and it colocalizes with PfMLH, a protein involved in mismatch repair. We have reported the schizont stage specific expression of PfDH60 helicase in a previous study [35] and PfMLH is also expressed in schizont stages [31]. The expression of PfUvrD in schizont stages further suggests that during this stage the parasite needs UvrD in order to correct the mismatches which might have ensued during the course of replication.

## Supporting Information

Table S1 **Substrates used.**
(DOCX)

Data S1 **Supplementary data and figures.** Figure S1: Structure modelling of PfUvrD using the Swiss Model program. Figure S2: The comparison of the structure with the template.
(PDF)
