# Supplementary material for: Plasmodium falciparum UvrD Helicase Translocates in 3′ to 5′ Direction, Colocalizes with MLH and Modulates Its Activity through Physical Interaction
Source: PLoS One. 2012 Nov 21;7(11):e49385. doi: 10.1371/journal.pone.0049385 (PMC3503981; doi:10.1371/journal.pone.0049385)
Supplement: Table S1 — Substrates used. (DOCX) [file pone.0049385.s001.docx]

**Table 1: Substrates used**

**Oligonucleotides used for labeling/Annealed DNA Name/Structure of the substrate**

1. 5’-(T)_15_GTTTTCCCAGTCACGAC(T)_15_-3’/M13 **Normal**

**
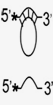
**

1. 5’-GTTTTCCCAGTCACGAC-3’/Complementary oligo **Blunt end**

1. 5’-TTCGAGCTCGGTACCCGGGGATCCTCTAGAGT-3’/M13 **A. 5’ to 3’ direction**

**B. 3’ to 5’ direction**
