# Supplementary material for: Plasmodium falciparum UvrD Helicase Translocates in 3′ to 5′ Direction, Colocalizes with MLH and Modulates Its Activity through Physical Interaction
Source: PLoS One. 2012 Nov 21;7(11):e49385. doi: 10.1371/journal.pone.0049385 (PMC3503981; doi:10.1371/journal.pone.0049385)
Supplement: Data S1 — Supplementary data and figures. Figure S1: Structure modelling of PfUvrD using the Swiss Model program. Figure S2: The comparison of the structure with the template. (PDF) [file pone.0049385.s002.pdf]

SWISS-MODEL Workspace

[myWorkspace] [login]

Workunit: P000079 PfUvrD - Overview

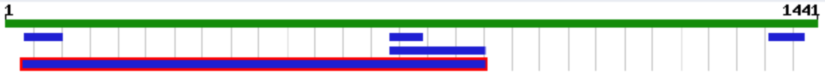

click on model bars

Models: [1] [2] [3] [4] [5]

Print/Save this page as

Model Summary

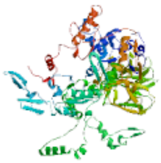

Model information:

Modelled residue range:	32 to 852
Based on template:	[3pjrA] (3.30 Å)
Sequence Identity [%]:	12.89
Evalue:	0.00e-1

Quality information: [details]
QMEAN Z-Score: -9.95

Warning: Low QMEAN Z-scores! Only membrane proteins or models of poor quality are expected to reach such low scores.

Quaternary structure information: [details]
Template (3pjr): MONOMER
Model built: SINGLE CHAIN

Ligand information: [details]
Ligands in the template: ATP: 1, DT: 4.
Ligands in the model: none.

logs: [Templates] [Alignment] [Modelling]
display model: as [pdb] - as [DeepView project] - in [AstexViewer]
download model: as [pdb] - as [Deepview project] - as [text]

Global Model Quality Estimation [+/-]

**QMEAN4 global scores:**			Local scores	
QMEANscore4	Estimated absolute model quality	Score components	Coloring by residue error	Residue error plot
0.19	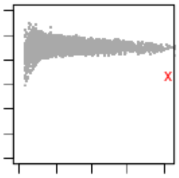 Z-Score: -9.95<br>Plot 1: [save png]<br>Plot 2: [save png]	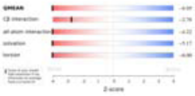 [save png]	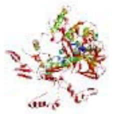	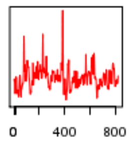 [save png]
			Coloring (all chains): [save jpg] [save pdb]	Energy profile: [save raw scores]

QMEAN4 global scores:

The QMEAN4 score is a composite score consisting of a linear combination of 4 statistical potential terms (estimated model reliability between 0-1). The pseudo-energies of the contributing terms are given below together with their Z-scores with respect to scores obtained for high-resolution experimental structures of similar size solved by X-ray crystallography:

Scoring function term	Raw score	Z-score
C_beta interaction energy	92.80	-2.76
All-atom pairwise energy	4590.11	-4.22
Solvation energy	56.08	-5.17
Torsion angle energy	86.01	-6.00
QMEAN4 score	0.187	-9.95

If you publish results from QMEAN, please cite the following paper:
Benkert P, Biasini M, Schwede T. (2011). "Toward the estimation of the absolute quality of individual protein structure models." Bioinformatics, 27(3):343-50.

Local Model Quality Estimation: Anolea / QMEAN / Gromos: [+/-]

anolea: ◉ on ○ off QMEAN: ◉ on ○ off gromos: ○ on ◉ off [show]

Figure S1

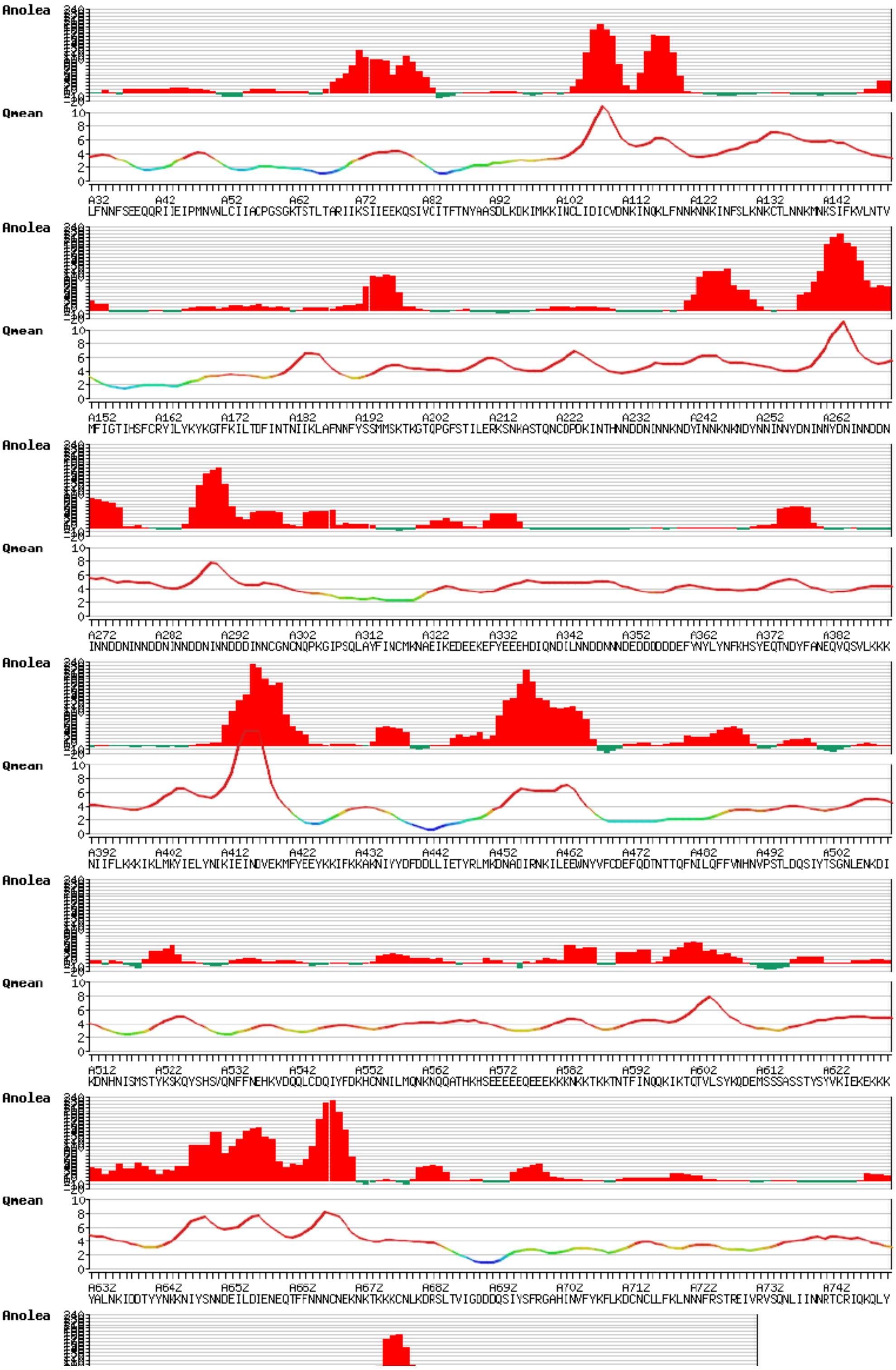

Download raw data: [Anolea Data] [QMEAN Data]

Alignment [+/-]

Modelling Log [+/-]

Template Selection Log [+/-]

Quaternary Structure Modeling Log [+/-]

Ligand Modeling Log [+/-]

References

Figure S1

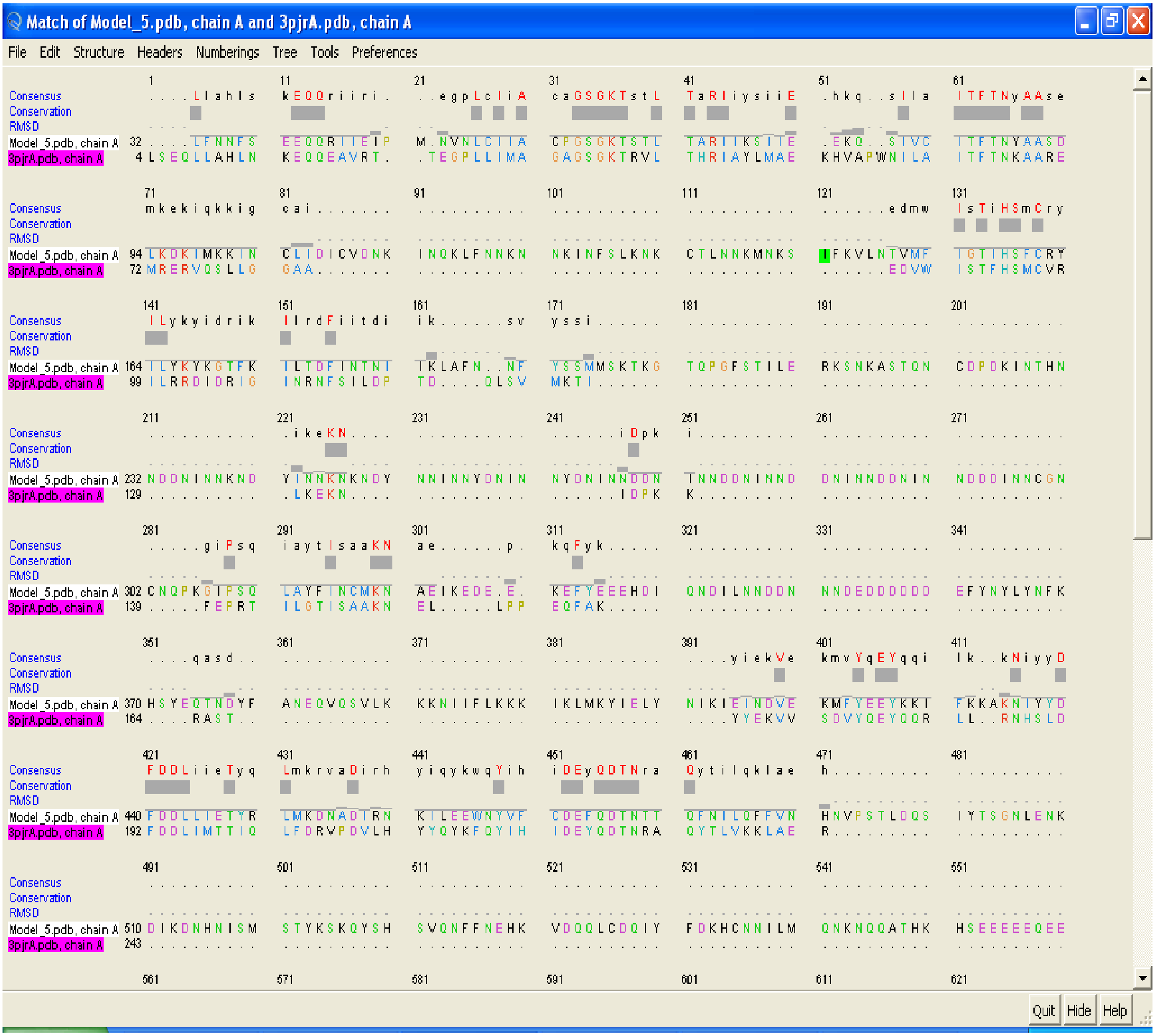

Match of Model_5.pdb, chain A and 3pjrA.pdb, chain A
File Edit Structure Headers Numberings Tree Tools Preferences
Consensus
Conservation
RMSD
Model_5.pdb, chain A
3pjrA.pdb, chain A
Quit
Hide
Help

Figure S2

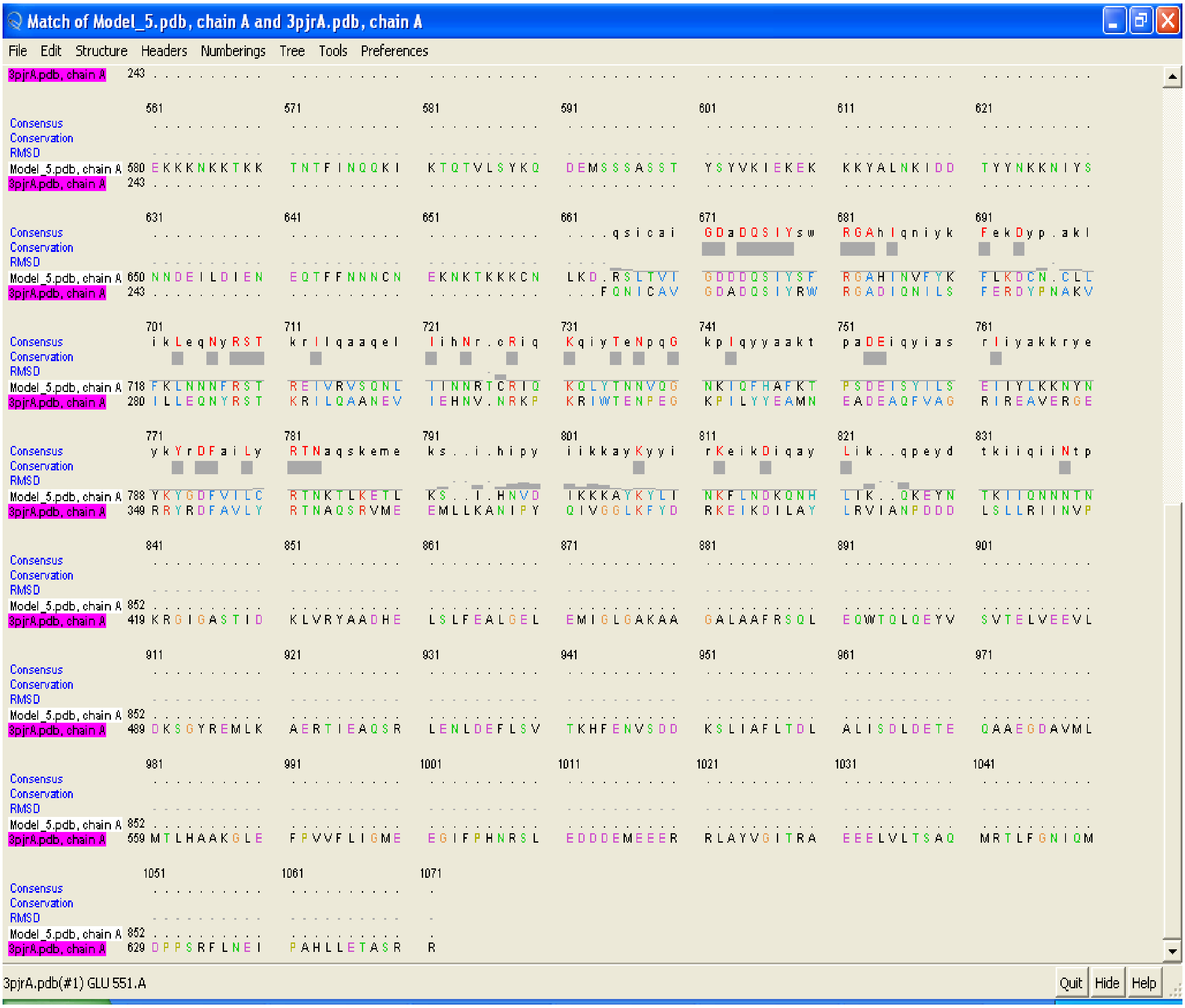
Match of Model_5.pdb, chain A and 3pjrA.pdb, chain A
File Edit Structure Headers Numberings Tree Tools Preferences
Consensus
Conservation
RMSD
Model_5.pdb, chain A
3pjrA.pdb, chain A
3pjrA.pdb(#1) GLU 551.A
Quit Hide Help

Figure S2
